# MSC-Derived Extracellular Vesicles Mitigate Ischemia-Induced Energetic Dysfunction During Ex Situ Perfusion of Rat Livers

**DOI:** 10.3390/antiox15070843

**Published:** 2026-07-04

**Authors:** Caterina Lonati, Michele Battistin, Andrea Carlin, Michela Ripolone, Francesco Fortunato, Valentina Fonsato, Alessia Brossa, Alberto Zanella, Giovanni Camussi, Daniele Eliseo Dondossola

**Affiliations:** 1 Center for Preclinical Research, Fondazione IRCCS Ca’ Granda Ospedale Maggiore Policlinico, Via Pace 9, 20122 Milan, Italy; 2 Department of Pathophysiology and Transplantation, University of Milan, 20122 Milan, Italy; 3 Neuromuscular and Rare Disease Unit, Fondazione IRCCS Ca’ Granda Ospedale Maggiore Policlinico, 20122 Milan, Italy; 4 Dino Ferrari Center, Department of Pathophysiology and Transplantation, University of Milan, 20122 Milan, Italy; 5 2i3t Incubatore per le Imprese, University of Torino, 10126 Turin, Italy; 6 Department of Molecular Biotechnology and Health Science, University of Torino, 10126 Turin, Italy; 7 Department of Medical Sciences, University of Torino, 10126 Turin, Italy; 8 General and Liver Transplant Surgery Unit, Fondazione IRCCS Ca’ Granda Ospedale Maggiore Policlinico, Via Francesco Sforza 35, 20122 Milan, Italy

**Keywords:** mesenchymal stromal cells-derived extracellular vesicles, ischemia/reperfusion injury, normothermic machine perfusion, mitochondrial dysfunction, succinate, FMN

## Abstract

Despite advances in liver machine perfusion (MP), ischemia–reperfusion injury (IRI) remains a major challenge in liver transplantation, with energetic stress and mitochondrial dysfunction recognized as key drivers of damage exacerbation. We investigated whether fractions enriched with extracellular vesicles (EVs) derived from mesenchymal stromal cells can preserve energetic homeostasis in rat livers undergoing normothermic MP (NMP). An established NMP rat model was used (n = 5 per group). After procurement, livers underwent NMP for 4 h, preceded or not by 30 min cold ischemia (CI). EVs (NMP + EVs and CI + NMP + EVs) or saline (NMP and CI + NMP) were randomly administered to the perfusion fluid. Perfusate samples were collected throughout the procedure, and biopsies were taken at the end of NMP. Ischemic livers exhibited succinate accumulation, flavin mononucleotide (FMN) release, activation of reverse electron transport, and adenosine triphosphate (ATP) depletion. EV treatment effectively counteracted these effects, restoring a metabolic profile comparable to that of non-ischemic livers. Moreover, EVs improved adenosine monophosphate/ATP ratios and prevented AMP-activated protein kinase activation, a key energy-stress sensor. Furthermore, EVs reduced oxidative stress markers, cell death mediators, and pro-inflammatory cytokines, indicating a broad cytoprotective and anti-inflammatory effect. These findings support the potential of EVs to preserve mitochondrial function, restore energy balance, and reduce inflammation, thereby improving liver cell viability during NMP.

## 1. Introduction

Ischemia/reperfusion injury (IRI) could lead to severe complications in liver transplantation, including primary nonfunction, graft dysfunction, and other post-transplant adverse events [1,2,3]. These detrimental effects are further exacerbated in grafts derived from extended criteria donors (ECD) or exposed to prolonged cold ischemia (CI) [3,4,5,6]. Given the growing use of such marginal organs to cope with organ shortage, reducing or preventing IRI represents a relevant perspective in transplantation research.

IRI is established through a complex chain of events, starting from ischemia-dependent depletion of energy substrates and culminating in sterile inflammatory activation upon reperfusion [7,8]. In particular, ATP depletion during ischemia is a critical determinant of cell fate, as low ATP content impairs ionic pump function, leading to cell swelling and Ca^2+^ accumulation, which in turn exerts a plethora of deleterious effects [9]. In the last decade, electron transport chain (ETC) aberrancies and reactive oxygen species (ROS) production have emerged as key contributors to tissue injury [10,11]. In particular, ROS generation and mitochondrial dysfunction trigger the opening of the mitochondrial permeability transition pore (mPTP), a key event in cell death activation [10,12]. ROS production during ischemia is primarily driven by the so-called reverse electron transport (RET) at complex I [10]. This latter phenomenon arises from the accumulation of succinate in ischemic cells and its subsequent rapid oxidation upon reperfusion [13].

In this context, machine perfusion (MP) offers two complementary advantages. First, it provides an unprecedented platform for investigating IR-induced processes in an isolated organ setting [14,15,16,17,18], thereby enabling to deepen the understanding necessary to develop novel intervention strategies. Second, the procedure itself represents a promising approach to mitigate these deleterious events before transplantation [19,20]. More specifically, a prominent advantage of MP lies in the possibility to deliver therapeutic agents directly into the isolated organ, providing a new therapeutic perspective for cell damage repair during the preservation phase [21,22,23], thereby opening new clinical research avenues for cellular repair prior to transplantation. In this regard, the addition of mesenchymal stromal cell (MSC)-based therapies to the MP perfusate was extensively proven to effectively reduce reperfusion injury [24,25].

Growing evidence indicated that the beneficial effects of stem cells primarily depend on the release of extracellular vesicles (EVs) [26], which contain genetic material, proteins, and organelles of the parent cell. Consistently, the therapeutic effectiveness of cell-free therapies in IRI resolution was documented both in vivo and ex situ [24,26,27,28,29,30,31,32]. Seminal studies showed that EV treatment can significantly support mitochondrial function [33,34,35,36]. Intriguingly, our research group demonstrated the ability of MSC-derived EV-enriched fractions to prevent ischemia-dependent ATP depletion in ex vivo lung perfusion [37] and in experimental liver MP [38]. This observation was associated with improved organ viability and function in both models.

These promising results support a more extensive investigation of the protective effect mediated by ex situ administration of EV-enriched fractions against the early deleterious events induced by ischemia and ex situ reperfusion. Therefore, the present study used a validated preclinical model of liver normothermic MP (NMP) [39,40] to investigate whether MSCs-derived EVs could preserve mitochondrial function and reduce energy stress. The main focus was on potential influences of EVs on ETC functionality and ATP production during the reperfusion phase. The advantages conferred by EV treatment on liver function, oxidative stress, and inflammatory status were likewise investigated.

## 2. Materials and Methods

### 2.1. Study Design

Twenty-five rats were randomly assigned to one of the following groups (n = 5 each): (1) Native, liver biopsies were collected from rats in resting conditions; (2) NMP, livers were procured from rat donors and immediately ex situ perfused for 4 h; (3) NMP + EVs, same as NMP group, except for the administration of MSCs-derived EVs during ex situ perfusion; (4) CI + NMP, livers were procured from rat donors, exposed to 30 min CI, then ex situ perfused for 4 h; (5) CI + NMP + EVs, same as CI + NMP group, except for the administration of MSCs-derived EVs during ex situ perfusion.

The EVs used in the present study were collected from 1 × 10^6^ human bone marrow MSCs, as described below, and were added directly into the perfusion fluid at 45 min after NMP. Treatments were performed using a blinded approach, with an equal volume of saline as vehicle.

### 2.2. Purification of EV-Enriched Fractions from Human Bone Marrow-Derived MSC Cultures

**Figure 1:**
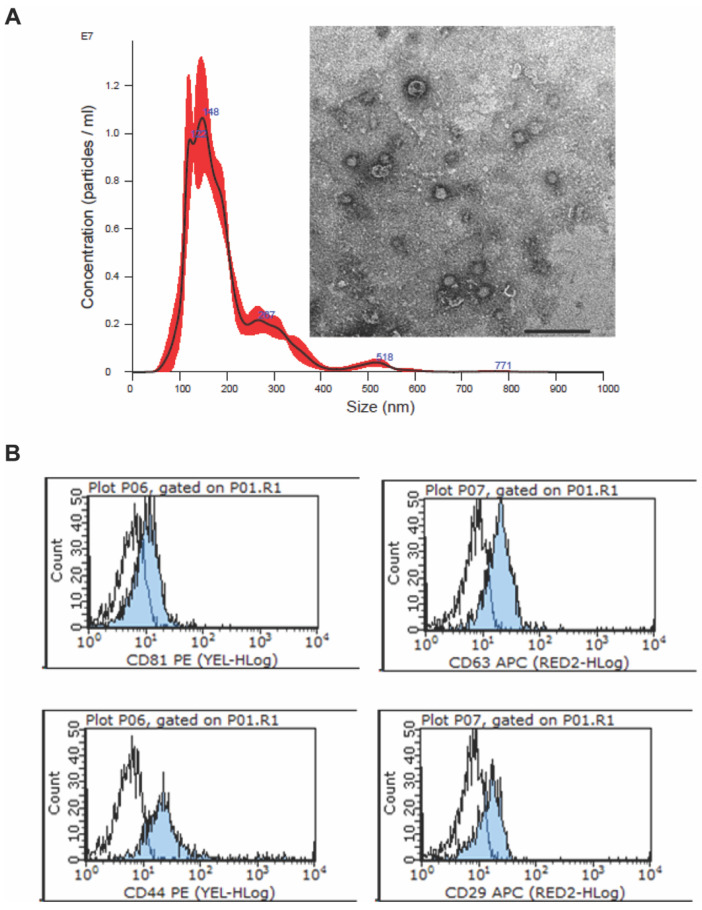
Characterization of MSC-EVs. (**A**) Representative micrograph of Nanoparticle tracking analysis (NTA) and Transmission electron microscopy (TEM) analysis, showing EV size distribution and integrity, respectively. respectively. Scale bar = 200 nm. (**B**) Representative FACS analysis showing the expression of both MSC (CD44, CD29) and exosomal markers (CD63 and CD81) (blue histogram), compared to isotype control (white histogram).

EVs were purified from 1 × 10^6^ MSCs sub-confluent human bone marrow-derived MSCs (Lonza Bioscience, Basel, Switzerland), and cultured in a MSC basal medium, as previously described [37,41]. Briefly, the supernatant was recovered after overnight culture in serum-free RPMI (Lonza Bioscience) and centrifuged for 15 min at 3000 *g* at 4 °C before being filtered using 0.22 µm pore filters to remove cell debris and apoptotic bodies. Supernatants were subjected to ultracentrifugation at 100,000 *g* for 2 h at 4 °C. EVs were then resuspended in RPMI supplemented with 1% dimethyl sulfoxide (Sigma-Aldrich, Merck KGaA, Darmstadt, Germany) and stored at −80 °C until subsequent use. For quantification and evaluation of EV size distribution, 5 µL of EVs were diluted in 1 mL PBS previously filtered using 0.1 µm pore filters and analyzed using a Nanosight LS300 system (Malvern Panalytical Ltd., Malvern, UK). EV integrity was confirmed by electron microscopy (Figure 1A), as previously described [41]. The expression of exosomal markers CD63 and CD81 and of the markers of the parent cells CD44 and CD29 was analyzed by flow cytometry analysis performed using a Guava^®^ system (Cytek Biosciences, Fremont, CA, USA) (Figure 1B), according to the reported protocol [41] (all reagents were from Beckton Dickinson, Franklin Lakes, NJ, USA) (Figure 1).

### 2.3. In Vivo Procedures

The procedures involving the use of laboratory animals were performed at the Center for Preclinical Research, under authorization number 456/2021. Animals received humane care in compliance with the European Union Directive 2010/63/EU and the Italian Legislative Decree 26/2014. Experiments were planned according to the Planning Research and Experimental Procedures on Animals: Recommendations for Excellence (PREPARE) guidelines and performed in compliance with the 3R principles. An “a priori” power analysis was carried out to estimate the minimum number of rats needed to reliably detect the expected effect size. Details on assumptions and calculations are provided in the Supporting Methods S1.1, as established by the “Animal Research: Reporting of In Vivo Experiments” (ARRIVE) guidelines.

Sprague–Dawley rats weighing 250–300 g were housed in a ventilated cage system at 22 ± 2 °C, 55 ± 10% humidity, on a 12 h dark/light cycle, and were allowed free access to food and water.

Methods for anesthesia induction, surgical procedures, and in situ flushing are described in Supporting Methods S1.2, as previously described [39]. In the CI groups, the cold preservation phase was performed by placing the organs in 100 mL of Celsior solution (IGL, Lissieu, France) at 4 °C.

### 2.4. Normothermic Machine Perfusion (NMP) and Hemodynamic Monitoring

Following back-table preparation, liver dynamic perfusion was performed according to the protocol previously reported by our research group [39], using a customized circuit equipped with a peristaltic pump, a membrane oxygenator, a heater, and pressure and temperature monitoring probes. The liver was placed in the organ chamber and connected to the perfusion system through the portal vein.

A description of the NMP protocol is provided in Supporting Methods S1.3. The system was primed with perfusion fluid supplemented with an oxygen carrier. The perfusion was maintained for 4 h and involved continuous pressure and temperature monitoring with hourly evaluation of perfusate acid-base balance, electrolytes, and metabolite concentration. Hemodynamics parameters were monitored throughout the procedure.

### 2.5. Sample Collection

During the first 5 min of NMP, the entire volume (20 mL) of the outflow perfusate was withdrawn from the vena cava; this sample was referred to as “wash-out”. Based on previously reported protocols, samples of the recirculating perfusate were collected hourly and adequately processed for subsequent biomolecular analysis [14].

Bile was collected into a test tube containing 200 µL of liquid paraffin to avoid bile-air contact.

Liver biopsies were obtained from the right median lobe at the end of NMP. Three specimens were immediately frozen in liquid nitrogen; one sample was frozen with isopentane, previously cooled with liquid nitrogen to preserve tissue morphology, while an additional biopsy was fixed in 2.5% glutaraldehyde in cacodylate buffer pH 7.4. Finally, one sample was used to assess wet-to-dry ratio (edema index) and one was formalin-fixed.

### 2.6. Perfusate Analyses

Biomarkers of liver cell damage, including aspartate aminotransferase (AST), alanine aminotransferase (ALT), and lactate dehydrogenase (LDH), were measured in perfusate samples, using routine automated clinical chemistry assays performed by the Clinical Chemistry Laboratory of Fondazione IRCCS Ca’ Granda Ospedale Maggiore Policlinico. Moreover, after processing following the procedure reported in the Supporting Methods S1.4, perfusates were used to assess the following parameters: succinate (Succinate Colorimetric Assay Kit, Sigma-Aldrich), flavin mononucleotide (FMN, according to the previously published protocols [18,42]), 8-hydroxy-2′-deoxyguanosine (8-OHdG) (DNA damage competitive ELISA, Thermo Fisher Scientific, Waltham, MA, USA), Caspase-cleaved keratin 18 (CK18) (Cusabio Technology LLC, Houston, TX, USA), Adenylate kinase (AK) (Lonza Bioscience), 3-hydroxybutyric acid and acetoacetic acid (Ketone Body Assay Kit, Sigma-Aldrich), and soluble proteins relevant to immune activation (Rat Cytokine/Chemokine Magnetic Bead Panel, EMD Millipore Corporation, Billerica, MA, USA). The panel included the following molecules: Chemokine C-C motif ligand 2/Monocyte Chemoattractant Protein-1, CCL2/MCP-1; CCL3/Macrophage Inflammatory Protein-1alpha, CCL3/MIP-1alpha; CCL5/regulated on activation, normal T cell expressed and secreted, CCL5/RANTES; C-X-C motif chemokine ligand 1/Cytokine-Induced Neutrophil Chemoatractant-1, CXCL1/CINC-1; CXCL5/Lipopolysaccharide-induced CXC chemokine, CXCL5/LIX; CXCL10/Interferon-gamma inducible Protein 10kDa, CXCL10/IP-10; Interleukin-4, IL-4; IL-6; IL-10; IL-18; Tumor Necrosis Factor-alpha, TNF-alpha; Vascular Endothelial Growth Factor, VEGF). Furthermore, the following molecules of human origin were evaluated: IL-4, IL-6, IL-10, IL-13, IL-1ra, IL-36-beta, Galectin-3 and CCL-2/MCP-1 (R&D Systems, Minneapolis, MN, USA).

All methods and reagents used for perfusate analysis are provided in the Supporting Methods S1.5.

### 2.7. Bile Analyses

After collection, bile was weighed to obtain the net hourly production, then 100 µL was analyzed with a gas analyzer to assess electrolyte composition.

### 2.8. Evaluations Performed on Liver Tissue Biopsies

Liver specimens were subjected to morphological evaluation and histochemical analysis to evaluate the presence and abundance of cytochrome c oxidase (COX) and succinate dehydrogenase (SDH) enzymes. Ultrastructural analysis by transmission electron microscopy (TEM) was likewise performed to explore mitochondrial morphology and integrity.

Moreover, tissue specimens were used to assess the activity of glycolytic enzymes and ETC complexes, as previously described [43].

Finally, liver biopsies were used to evaluate the tissue energetic pool by means of a luciferase-based method (Enliten ATP Assay System, Promega, Madison, WI, USA) and High-performance liquid chromatography (HPLC). The activation of AMP-activated protein kinase (AMPK) signaling was explored by Western blot analysis.

All methods and reagents used are described in the Supporting Methods S1.6.

### 2.9. Statistical Analysis 

Data are presented as mean ± standard error of the mean (SEM) or median [25th–75th percentile] when appropriate. Differences across experimental groups were investigated using one-way analysis of variance (ANOVA) or two-way repetitive measures (RM) ANOVA, followed by Tukey’s post hoc test for multi-comparison procedures. Non-normally distributed data were either rank-transformed before ANOVA or non-parametric tests were applied to investigate differences. A probability value of *p* < 0.050 was considered significant. Statistical tests were performed using SigmaStat software 11.0 (Systat Software Inc., San Jose, CA, USA), JMP Pro 17.2.0 (JMP Statistical Discovery LLC, SAS Campus Drive, NC, USA), and Prism version 9.4.1 (GraphPad Software LL, La Jolla, CA, USA).

## 3. Results

### 3.1. Liver Functional Assessment and Morphological Evaluation

No differences in portal pressures and vascular resistances were observed across experimental groups (*p* = 0.845 and *p* = 0.954, respectively; Supplementary Table S1).

Perfusate gas analysis revealed some differences between ischemic and non-ischemic livers, which are reported in detail in the Supplementary Table S2.

Total bile output was significantly lower in livers exposed to ischemia compared to organs immediately perfused after procurement (CI + NMP vs. NMP: *p* = 0.016; Supplementary Figure S1). However, EV treatment was associated with no significant changes in bile production (CI + NMP + EV vs. CI + NMP: *p* = 0.099). Bile gas analysis is reported in Supplementary Table S3.

Liver edema index was similar across experimental groups (CI + NMP + EVs: 3.22 ± 0 vs. CI + NMP: 3.28 ± 0.01).

Histological analysis revealed no morphological alterations in the perfused tissues compared to the Native group (Supplementary Figure S2).

### 3.2. Effects Exerted by EV Treatment on Mitochondrial Oxidative Phosphorylation and ETC Function

**Figure 2:**
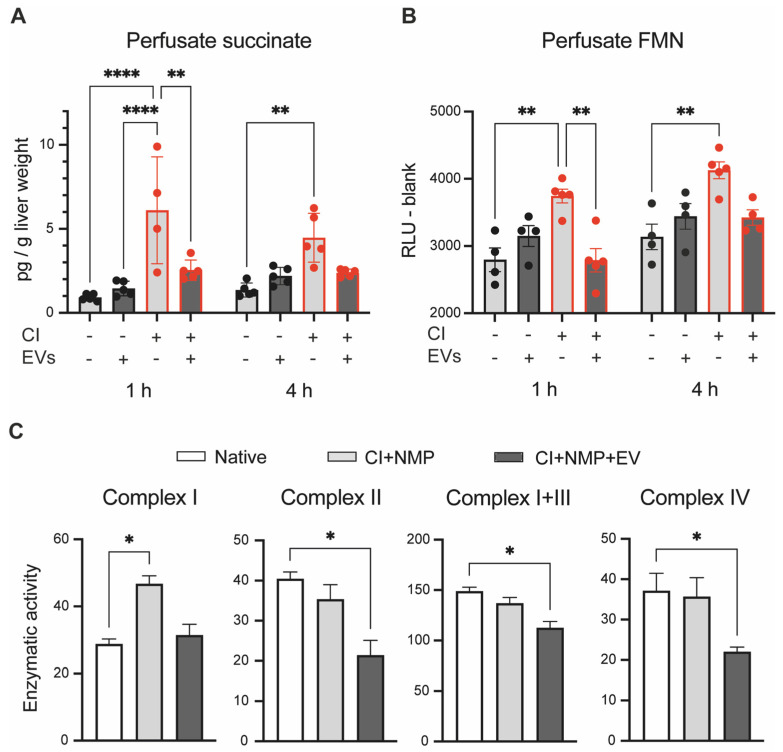
Effects exerted by ex situ administration of MSC-derived EVs on mitochondrial function of livers exposed to ischemia. (**A**) EVs reversed succinate accumulation induced by ischemic challenge. Perfusate succinate was determined by enzymatic assay. Bars denote mean ± SEM, n = 5 biologically independent replicates; Two-way ANOVA, Tukey’s post hoc test. CI + NMP vs. NMP: *p* < 0.0001 at 1 h and *p* < 0.0001 at 4 h; CI + NMP + EV vs. CI + NMP: *p* = 0.002 at 1 h and *p* = 0.137 at 4 h. Asterisk legend: ** *p* < 0.01; **** *p* < 0.0001 (**B**) EVs treatment was associated with a reduced release of FMN, a component of mitochondrial complex I. Bars denote mean ± SEM, n = 5 biologically independent replicates; Two-way ANOVA, Tukey’s post hoc test. CI + NMP vs. NMP: *p* = 0.003 at 1 h and *p* = 0.002 at 4 h; CI + NMP + EV vs. CI + NMP: *p* = 0.001 at 1 h and *p* = 0.053 at 4 h. (**C**) EVs influenced the activity of the mitochondrial complexes involved in the electron transfer chain (ETC). Enzymatic activity was assessed in liver tissue homogenates according to previously reported protocols [43]. Data are reported as pmol/min/mg tissue and normalized to citrate synthase activity. Bars denote mean ± SEM, n = 3 biologically independent replicates; One-way ANOVA, Tukey’s post hoc test. Complex I (NADH: ubiquinone oxidoreductase): CI + NMP vs. Native: *p* = 0.018; Complex II (Succinate CoQ reductase): CI + NMP + EV vs. CI + NMP: *p* = 0.028; Complex I + III (NADH cytochrome C reductase): CI + NMP + EV vs. CI + NMP: *p* = 0.018; Complex IV (Cytochrome C oxidase): CI + NMP + EV vs. CI + NMP: *p* = 0.031. Asterisk legend: *p* values: * *p* < 0.05. Abbreviations: CI, cold ischemia; EV, extracellular vesicles; FMN, flavin mononucleotide; NMP, normothermic machine perfusion.

Perfusate analysis revealed a dramatic increase in succinate in samples collected from livers exposed to CI compared to those subjected to NMP alone (Figure 2A; *p* < 0.0001 at 1 h and *p* = 0.005 at 4 h). Remarkably, EV administration was associated with no rise of succinate (Figure 2A). More specifically, the CI + NMP + EVs group exhibited a similar succinate concentration to that observed in livers perfused immediately after procurement (CI + NMP + EVs vs. NMP: *p* = 0.817 at 1 h), while it was significantly lower than that detected in untreated livers (CI + NMP + EVs vs. CI + NMP: *p* = 0.002 at 1 h).

Perfusate FMN showed a similar trend (Figure 2B). In fact, while this metabolite significantly increased in the CI + NMP group (CI + NMP vs. NMP: *p* = 0.003 at 1 h and *p* = 0.002 at 4 h of NMP), EVs-treated livers had comparable succinate concentrations to that of the NMP group (CI + NMP + EVs vs. NMP: *p* = 0.999 at 1 h and *p* = 0.904 at 4 h).

Based on these results and the higher LDH activity observed in livers exposed to the ischemic challenge compared to the Native group and to livers immediately perfused after procurement (Supplementary Figure S3), the investigation of mitochondrial function was focused exclusively on livers exposed to ischemia, while native livers were used as reference controls.

Immunohistochemistry revealed similar staining for COX and SDH compared to the Native group (Supplementary Figure S4). Conversely, evaluation of ETC complex activity in liver biopsies disclosed significant differences across the experimental groups (Figure 2C). An increased activity at Complex I (NADH: ubiquinone oxidoreductase) was detected in livers exposed to CI (CI + NMP vs. Native: *p* = 0.018), whereas no significant differences were observed between the CI + NMP + EVs group and native livers (*p* = 0.842). Intriguingly, compared to the Native group, livers exposed to ischemia and then treated with EVs exhibited a lower activity of Complex II (Succinate CoQ reductase, *p* = 0.028), Complex I + III (NADH cytochrome C reductase, *p* = 0.018), and Complex IV (Cytochrome C oxidase, *p* = 0.031). A consistent pattern was observed for SDH (Supplementary Figure S5, *p* = 0.077) and Complex II + III (Succinate cytochrome C reductase; Supplementary Figure S6, *p* = 0.074), though there was no statistical significance. No changes in NADH dehydrogenase activity were revealed across experimental groups (Supplementary Figure S7, *p* = 0.615). Finally, assessment of Pyruvate kinase (PK) further confirmed poor recovery of mitochondrial function in untreated livers, which exhibited a higher activity compared to the Native group (Supplementary Figure S8, *p* = 0.027). In contrast, the CI + NMP + EVs group showed a similar PK activity to that of native livers (Supplementary Figure S9, *p* = 0.930 vs. Native).

### 3.3. Electron Microscopy Analysis to Assess Mitochondrial Status and Structure

**Figure 3:**
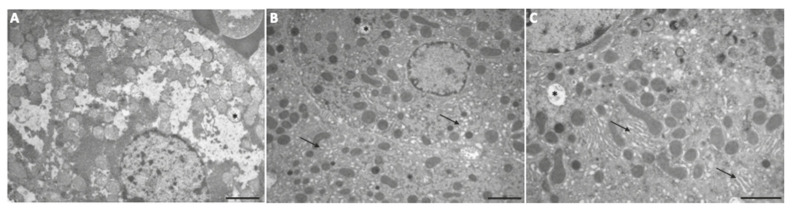
Ultrastructural analysis of liver biopsies. Representative Transmission electron microscopy (TEM) images of the different experimental groups: (**A**) Native; (**B**) CI + NMP; (**C**) CI + NMP + EV. Mitochondria quantity and morphology were similar across all groups. Rarely, dilated mitochondria with cristae rarefaction and matrix loss were observed (asterisks). A notable difference was observed in the ER, which appeared more abundant and organized in the Native group and occasionally dilated in groups exposed to CI (arrows). Scale Bar: (**A**,**B**): 2.5 µm; (**C**): 2 µm.

TEM ultrastructural analyses were performed to explore mitochondrial morphology and integrity in EVs-treated livers (Figure 3A–C). Compared to the Native group, no changes in mitochondria quantity and morphology were observed in perfused livers. Occasionally, we observed dilated mitochondria displaying cristae rarefaction and matrix loss in all the experimental groups. Of note, there was a marked reduction in the endoplasmic reticulum (ER), which was less prominent in the NMP groups. Moreover, livers exposed to CI showed a dilated ER compared to the Native group.

### 3.4. EVs Restored Liver Cell Energy Pool, Preventing the Activation of AMPK Signaling

**Figure 4:**
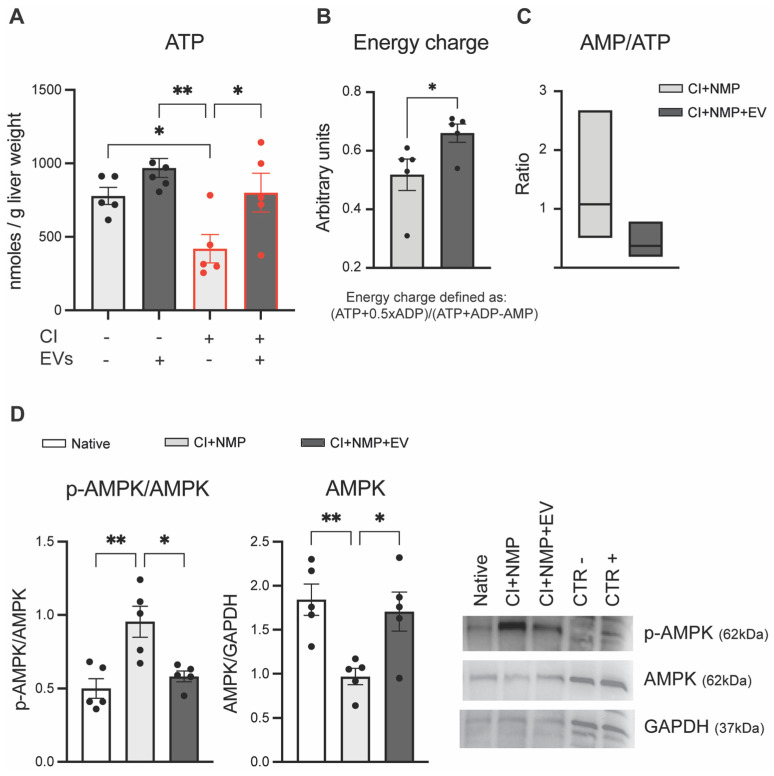
MSC-derived EVs recovered liver cell energy metabolism and prevented the activation of AMPK signaling in organs exposed to ischemia. (**A**) EV treatment restored tissue ATP content, reversing the decrease in energy pool elicited by ischemic damage. Tissue ATP content was measured by bioluminescent assay in liver tissue homogenates and normalized to liver wet weight. Bars denote mean ± SEM, n = 5 biologically independent replicates. Two-way ANOVA, Tukey’s post hoc test. CI + NMP vs. NMP + EV: *p* = 0.002; CI + NMP + EV vs. CI + NMP: *p* = 0.042. Asterisk legend: *p* values: * *p* < 0.05; ** *p* < 0.01 (**B**) EVs mitigated the depletion in energy charge elicited by ischemia. ATP, ADP, and AMP were determined in liver biopsies by High-performance liquid chromatography (HPLC). Total energy charge was calculated according to a previously validated formula [44]. Bars denote mean ± SEM, n = 5 biologically independent replicates. Mann–Whitney test: CI + NMP + EV vs. CI + NMP: *p* = 0.047. Asterisk legend: *p* values: * *p* < 0.05 (**C**) EV-treated livers exhibited a lower AMP/ATP ratio compared to the CI + NMP group. Floating bars illustrate minimum value, mean value, and maximum value. Mann–Whitney test: CI + NMP + EV vs. CI + NMP: *p* = 0.055. (**D**) The ischemia-induced activation of AMPK signaling was prevented by EV ex situ administration. AMPK phosphorylation was investigated by immunoblotting in tissue homogenates. Bars denote mean ± SEM, n = 5 biologically independent replicates; One-way ANOVA, Tukey’s post hoc test. Ratio phospho-AMPK on AMPK: CI + NMP vs. Native: *p* = 0.048, CI + NMP + EV vs. CI + NMP: *p* = 0.028. AMPK normalized on GAPDH: CI + NMP vs. Native: *p* = 0.012, CI + NMP + EV vs. CI + NMP: *p* = 0.015. Asterisk legend: *p* values: * *p* < 0.05; ** *p* < 0.01. Representative immunoblotting images are shown in the right panel. Abbreviations: CI, cold ischemia; EV, extracellular vesicles; AMP, adenine monophosphate; ADP, adenine diphosphate; ATP, adenine monophosphate; AMPK, AMP-activated protein kinase; NMP, normothermic machine perfusion.

Cell energetic status was investigated in liver tissue biopsies following two different methodological approaches, including measurement of overall ATP content by means of a luciferase-based assay and subsequent HPLC analysis to assess ATP and ATP-derived metabolites. As shown in Figure 4A, while the energetic pool dropped in untreated livers exposed to the ischemic challenge (CI + NMP vs. NMP + EVs: *p* = 0.002), EV treatment was associated with reduced ATP depletion (CI + NMP + EVs vs. CI + NMP: *p* = 0.042).

This observation was consistent with the data provided by HPLC of liver homogenates (Figure 4B). In particular, the CI + NMP + EVs group exhibited the following proportion of purine nucleotides: ATP 48.33% (vs. 38.14% in the CI + NMP group), ADP 35.57% (vs. 26.93%), and AMP 16.10% (vs. 34.93%). A higher inosine content was likewise observed in the CI + NMP + EV group compared to untreated livers (Supplementary Figure S9, *p* < 0.050). Total energy charge was 0.66 ± 0.03 in EVs-treated livers vs. 0.51 ± 0.05 in the CI + NMP group (Figure 4B, *p* = 0.047). Of note, the higher AMP content observed in the CI + NMP + EV group (*p* = 0.032) reflected a different, although not significant, AMP/ATP ratio (Figure 4C, *p* = 0.055).

Based on this latter result, we investigated whether ischemic challenge triggered the AMPK signaling and the potential effects of EVs on this key regulator of cellular energy homeostasis. As illustrated in Figure 4D, an increased content of phospho-AMPK was observed in the CI + NMP group compared to native livers (*p* = 0.003). In contrast, EV-treated livers showed a reduced degree of AMPK phosphorylation (*p* = 0.011 vs. CI + NMP; *p* = 0.725 vs. Native). On the other hand, non-phosphorylated AMPK resulted significantly lower in the CI + NMP group compared to both the Native (*p* = 0.010) and CI + NMP + EV (*p* = 0.027) groups.

### 3.5. Influence of EV Treatment on Cell Viability, Function, and Oxidative Damage

**Figure 5:**
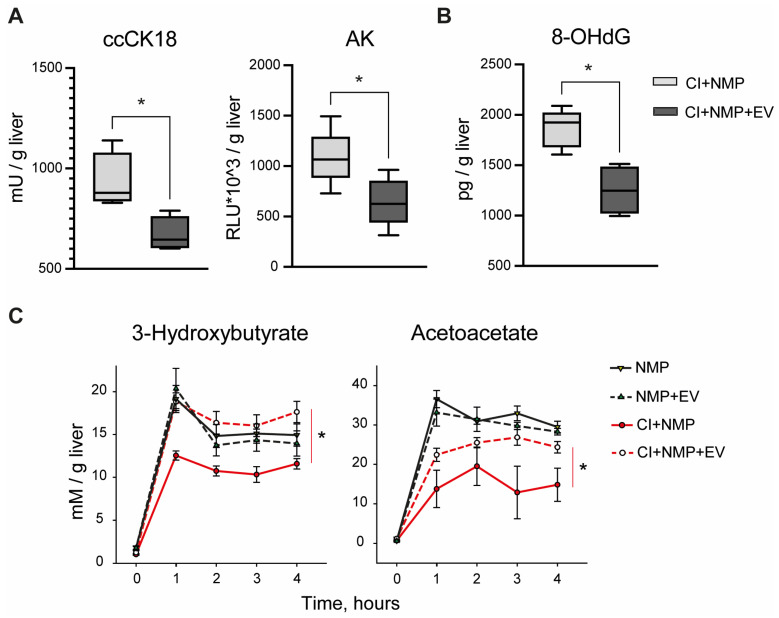
Protective effects exerted by MSC-derived EVs in livers exposed to ischemia. (**A**) Total release of cell death biomarkers was lower in the CI + NMP + EV group compared to untreated livers. Boxes denote median (25th–75th), n = 5 biologically independent replicates; Mann–Whitney test. Caspase-cleaved CK18: CI + NMP + EV vs. CI + NMP: *p* = 0.029. AK: CI + NMP + EV vs. CI + NMP: *p* = 0.032. Asterisk legend: *p* values: * *p* < 0.05. (**B**) EVs reduced oxidative stress. EV-treated livers showed a decreased release of 8-OHdG, a well-recognized indicator of oxidative DNA damage. Boxes denote median (25th–75th), n = 5 biologically independent replicates; Mann–Whitney test, CI + NMP + EV vs. CI + NMP: *p* = 0.016. Asterisk legend: *p* values: * *p* < 0.05. (**C**) Assessment of ketone body production. EV treatment restored both 3-hydroxybutyrate (*p* < 0.050) and acetoacetate (*p* < 0.050) production, which was in contrast affected in ischemic untreated livers. Points denote mean ± SEM, n = 5 biologically independent replicates; Two-way ANOVA, Tukey’s post hoc test. Asterisk legend: *p* values: * *p* < 0.05. Abbreviations: ccCK18, caspase-cleaved cytokeratin 18; AK, Adenylate kinase; 8-OHdG, 8-hydroxy-2′-deoxyguanosine; CI, cold ischemia; EV extracellular vesicles; NMP, normothermic machine perfusion.

The potential effects of EVs on cell viability and oxidative balance were assessed by measuring the release of cytolysis biomarkers and free radical adducts over the NMP procedure. Compared to the CI + NMP group, EV-treated livers showed a lower release of caspase-cleaved CK18 and AK, reflecting a reduced cell lysis subsequent to apoptosis and unspecified cell death (Figure 5A, *p* = 0.029 and *p* = 0.032). A decreased perfusate concentration of clinical biomarkers of hepatonecrosis was likewise observed (Supplementary Figure S10, AST: *p* < 0.001 at 4 h; ALT: *p* = 0.002 at 4 h; LDH: *p* = 0.008 at 4 h). The analysis of K+ perfusate concentration further supported this protection conferred by EV treatment (Supplementary Figure S11, *p* = 0.007).

With regard to ROS production, compared to the CI + NMP group, EVs-treated livers showed a reduced concentration of 8-OHdG, a well-recognized indicator of oxidative DNA damage (Figure 5B, *p* = 0.016).

Further evidence of oxidative stress modulation was provided by the assessment of ketone body concentration in the perfusate (Figure 5C). In fact, a restored production of both β-hydroxybutyrate (*p* < 0.050 vs. CI + NMP) and acetoacetate (*p* < 0.050 vs. CI + NMP) was observed in EV-treated livers, which exhibited similar levels to those of organs not exposed to ischemia.

### 3.6. EVs Were Associated with Significant Changes in the Release of Inflammation Mediators

**Figure 6:**
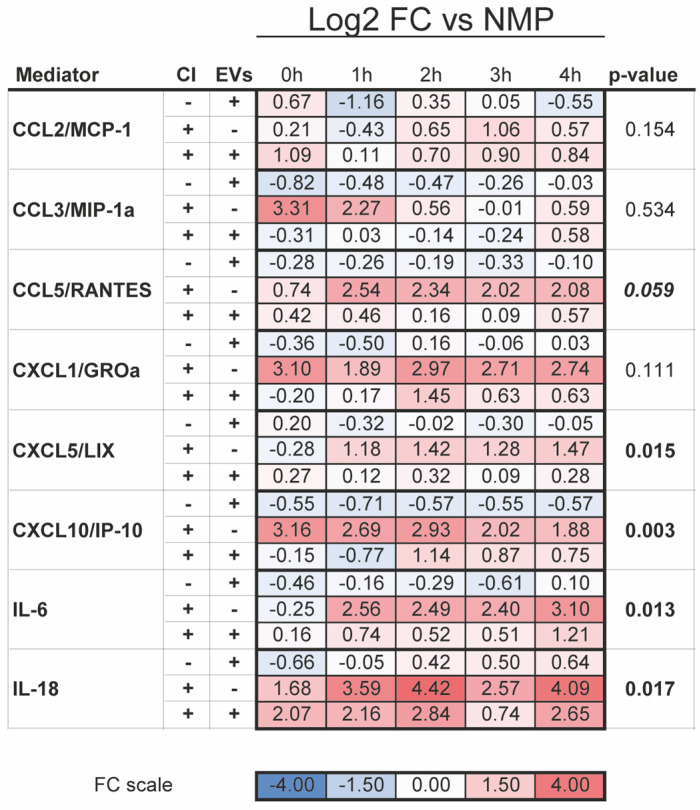
Perfusate inflammatory profile. Influence of MSC-derived EVs on the release of inflammatory mediators into the perfusate. The heatmap shows the log2-transformed fold change in mediator concentrations in the NMP + EV, CI + NMP, and CI + NMP + EVs groups, calculated relative to the values measured in the NMP group. Two-way RM ANOVA, Tukey’s post hoc test; *p*-values were FDR-adjusted. Abbreviations: CI, cold ischemia; EVs, extracellular vesicles; FC, Fold change; NMP, normothermic machine perfusion.

Next, perfusate mediator profiling was performed to explore whether EVs could mitigate the sterile inflammatory response associated with IRI. As shown in Figure 6, the CI + NMP + EV group showed lower perfusate concentration of the neutrophil-recruiting factor CXCL5/LIX (*p* = 0.015) and of the broader chemokine CXCL10/IP-10 (*p* = 0.003). The pro-inflammatory cytokine IL-6 (*p* = 0.013) and IL-18 (*p* = 0.017) were likewise decreased in livers exposed to EV treatment compared to untreated organs. On the other hand, there was no significant difference in the concentration of molecules typically involved in the later phases of sterile inflammation, including CCL5/RANTES (*p* = 0.059), CCL2/MCP-1 (*p* = 0.154), and CCL3/MIP1alpha (*p* = 0.534).

Of interest, analysis of selected molecules of human origin indicated that livers exposed to ischemic injury were associated with a greater utilization of the anti-inflammatory molecules IL-1ra (*p* = 0.006), IL-10 (*p* = 0.012), and Galectin-3 (*p* = 0.034) (Supplementary Figure S12).

## 4. Discussion

The present research demonstrates that ex situ administration of MSC-derived EV-enriched fractions induces a broad protection of cell metabolism in livers exposed to ischemia followed by reperfusion injury. Specifically, EV treatment reversed ischemia-dependent succinate accumulation, restored mitochondrial ETC function, and prevented ATP depletion. These remarkable effects were associated with a general improvement in liver cell viability, as evidenced by reduced release of oxidative stress and cell damage markers, restored ketone body production, and lower perfusate inflammatory mediators.

Mitochondrial energy stress and ROS generation are central drivers in the establishment of IRI [7,8,12,45]. In the context of organ transplantation, a better mitochondrial function during clinical MP was associated with improved transplant outcome [44], highlighting the profound impact of these processes on graft quality. Therefore, targeting mitochondrial metabolism appears as a promising therapeutic approach to enhance transplantation success [46].

Although the efficacy of MSC-derived EVs in ameliorating liver IRI has been the subject of many studies [31,32], few researchers have focused on their potential effects on energetic stress. Moreover, only partial success in the setting of liver MP has been achieved, mainly due to heterogeneity in models of ex situ preservation and perfusion [30,31]. Thanks to the use of a standardized NMP protocol [39], the present research provides substantial findings attesting that EVs administered at reperfusion can effectively mitigate mitochondrial dysfunction, conferring a benefit against the initial deleterious events elicited by ischemia. Of note, our evidence suggests that the protection of mitochondrial function conferred by MSC-derived EVs does not solely rely on the transfer of viable mitochondria to injured cells [33,34,35,36]. Indeed, the EV-enriched fractions used in the present research consisted of vesicles within the 100–200 nm size range, whereas intact mitochondria are larger organelles. Therefore, our data raise the possibility that additional mechanisms, beyond mitochondrial transfer, could contribute to the metabolic recovery induced by EV treatment in IRI.

The present study demonstrates for the first time that MSC-derived EV treatment can prevent succinate accumulation, a crucial aberration driving the onset of reperfusion injury [13]. In fact, succinate buildup during ischemia triggers mitochondrial ROS generation at Complex I, exacerbating oxidative damage upon reperfusion [10,13]. Notably, it has been previously reported that pharmacological prevention of succinate rise was sufficient alone to improve IRI outcomes in models of heart attack and stroke [13], further demonstrating the key role of this biological event in ischemic damage. Although the precise mechanism by which EV treatment normalized succinate tissue levels was not investigated in the present study, previous studies analyzing MSCs-derived EV cargo described the presence of mitochondria-associated proteins [36,47,48]. These findings were confirmed in a recent experimental work and meta-analysis, where substantial amounts of proteins related to the ETC were detected in vesicles isolated using different methods [49]. The delivery of proteins involved in mitochondrial metabolic pathways to injured cells has already been indicated as a potential mechanism contributing to cellular bioenergetic restoration [33,36]. Nevertheless, the presence of TCA-cycle enzymes was not directly evaluated in our EV preparations and requires targeted investigation in future studies.

The positive effect on succinate was associated with modulation of the activity of ETC complexes. Indeed, EV therapy prevented the activation of reverse electron flow through Complex I, which, in contrast, was induced in untreated livers. RET activation is a key event in IRI establishment, as it amplifies ROS production and triggers cell death in different organs [7,10]. Blocking RET during reperfusion has been shown to prevent oxidative stress, mPTP opening, and cell death in rat models of IRI [7]. In addition to Complex I modulation, EV-treated livers exhibited reduced activation of downstream ETC components, a phenomenon previously associated with mitigation of reperfusion injury [7].

The reduced dissociation of the FMN prosthetic group from Complex I, evaluated by measuring its perfusate concentration, confirmed that EV therapy effectively reduced RET activation. In the clinical setting, perfusate FMN concentration provides valuable information on mitochondrial stress [10] and on the extent of ischemic damage [42]. The predictive strength of this biomarker was validated in a recent large-cohort international study, which demonstrated its ability to assess graft quality before transplantation [50]. In line with these observations, livers subjected to EV treatment showed reduced oxidative stress and improved cytolysis biomarker profile. These compelling findings support the therapeutic relevance of succinate clearance and preservation of Complex I integrity, providing new insights into the efficacy of MSC-EVs for liver ex situ repair before transplantation.

Another compelling finding of the present research was the ability of EVs to preserve cellular energy balance, as indicated by improved ATP levels and reduced ATP breakdown into adenine nucleotides in EV-treated livers. The lower AMP/ATP ratio observed in livers treated with EVs was associated with reduced activation of AMPK, an energy-sensing kinase phosphorylated under energy-depleted conditions [51]. This observation suggests that EV-mediated preservation of cellular energetic homeostasis was sufficient to prevent the activation of energy-stress signaling pathways. The increased ketone body production further supports the restoration of mitochondrial metabolic activity in EV-treated livers.

Finally, beyond the protection of cell metabolism, EVs mediated additional cytoprotective effects, as demonstrated by the reduced release of oxidative stress, cell damage, and inflammatory markers in EV-treated livers relative to controls. These detrimental processes are recognized downstream consequences of mitochondrial dysfunction during IRI [9,46]. Consistently, preserving mitochondrial integrity can prevent the release of mitochondrial alarmins that promote inflammatory activation [12,46]. Moreover, the immunomodulatory properties of EVs could also depend on the release of their protective cargo [26,32], as suggested by the higher uptake of EVs-derived anti-inflammatory mediators IL-1ra and IL-10 in livers exposed to ischemia compared to non-ischemic organs.

We acknowledge some limitations in the present study. First, only male rats were used, since at the time the experiments were performed, no sex-based differences were reported in the context of short-term organ ex situ perfusion. Therefore, the inclusion of female animals would have been in conflict with the Reduction principle. Second, livers were exposed to a relatively short CI time. This methodological choice was selected to investigate the early energetic alterations induced by IR and to determine whether EV treatment could modulate these initial pathogenic events. Moreover, the protocol adopted in this study enabled us to minimize potential confounding factors related to a more pronounced ischemic injury, thereby enhancing the precision of our NMP platform in addressing the primary study objectives. In line with the results of our previous studies [16,38], the protocol for rat liver NMP reproduced the key detrimental biological events induced by IR, including energetic dysfunction, oxidative stress, and inflammatory activation. This being said, we acknowledge that the present model does not recapitulate the complexity and severity of injury encountered in the clinical setting. Future studies based on extended CI times will be necessary to further explore the MSC-EV efficacy under different ischemia durations. Third, only a single EV dose and administration time were investigated in the present study. Therefore, no conclusions can be drawn regarding dose–response relationships or the therapeutic window of EV administration. Fourth, we highlight some limitations in the methods used to investigate mitochondrial complex function. In fact, enzymatic activity was assessed by adding the necessary metabolic substrates to the isolated mitochondria in vitro [43]. Since reversible ischemic injury does not alter the protein structure of mitochondrial complexes, but rather it affects the metabolite environment required for oxidative phosphorylation, the addition of the missing substrates could have restored the homeostatic balance. Therefore, the methodological approach adopted in this study likely failed to fully capture the aberrancies induced by ischemia in the liver tissue, potentially leading to an underestimation of the differences between treated and untreated livers. Finally, concerns could be raised regarding the potential immunological effects of administering human-derived EVs in a rat model. However, perfusate profiling revealed an improved inflammatory phenotype in ischemic livers subjected to EV-treatment compared to untreated controls. Therefore, although cross-species immune interactions cannot be excluded, our data indicated no adverse immunological responses within the present experimental setting.

In conclusion, the present study provides multiple lines of evidence supporting the ability of an ex situ therapy based on MSCs-EVs-enriched fractions to mitigate the energetic derangements induced by ischemia. In particular, to the best of our knowledge, this is the first research demonstrating that EV treatment can prevent succinate accumulation and mitigate the mitochondrial alterations associated with reperfusion injury in liver exposed to IR. Given that early mitochondrial dysfunction has been linked to poor graft quality in liver transplantation [44,50], these findings support the rationale for targeting acute energetic stress during NMP. Our results encourage future investigations to fully harness EV therapeutic potential in ex situ perfusion settings under different clinical protocols, with the aim of improving overall graft outcome. Dedicated studies will be required to further optimize this therapeutic approach, by the identification of ideal EV administration strategies, manufacturing procedures, and MSC priming protocols [52,53,54].

## Data Availability

The data that supports the findings of this study are available in the Supplementary Material of this article.

## Supplementary Materials

The following supporting information can be downloaded at: https://www.mdpi.com/article/10.3390/antiox15070843/s1. Supporting methods: S1.1. Sample size calculation; S1.2. Anesthesia, surgery, in situ perfusion, and rat liver procurement; S1.3. Rat liver normothermic perfusion; S1.4. Perfusate sample processment; S1.5. Analysis of perfusate samples; S1.5.1. Succinate; S1.5.2. Flavin mononucleotide (FMN); S1.5.3. 8-hydroxy-2′-deoxyguanosine (8-OHdG); S1.5.4. Cell death markers: Caspase-cleaved keratin 18 (CK18) and Adenylate kinase (AK); S1.5.5. Ketone bodies; S1.5.6. Molecules relevant to inflammation and its resolution; S1.5.7. Human IL-4, IL-6, IL-10, IL-13, IL-1ra, IL-36-beta, Galectin-3 and CCL-2/MCP-1; S1.6. Evaluations performed on rat liver biopsies; S1.6.1. Wet-to-Dry Ratio; S1.6.2. Histological and histochemical analysis; S1.6.3. Ultrastractural analysis by Transmission electron microscopy (TEM); S1.6.4. Activity of glycolysis enzymes and ETC complexes; S1.6.5. ATP content assessment; S1.6.6. ATP derivatives by High-performance liquid chromatography (HPLC); S1.6.7. Western blot to assess AMP-activated protein kinase (AMPK) signaling. Supplementary figures: Figure S1. Bile output; Figure S2. Morphological evaluation a of liver biopsies; Figure S3. Activity of Lactate dehydrogenase (LDH); Figure S4. Immunohistochemistry of liver biopsies; Figure S5. Activity of Succinate dehydrogenase (SDH); Figure S6. Activity of mitochondrial Complex II + III; Figure S7. Activity of NADH dehydrogenase; Figure S8. Activity of Pyruvate kinase; Figure S9. Inosine content in liver tissue homogenates; Figure S10. Perfusate concentration of transaminases and LDH; Figure S11. Perfusate concentration of K+ during the NMP procedure; Figure S12. Perfusate concentration of mediators of human origin. Supplementary tables: Table S1: Hemodynamics monitoring during NMP; Table S2: Gas-analysis of perfusate samples; Table S3: Bile gas-analysis.

## Abbreviations

Abbreviations (alphabetical order): 8-OHdG, 8-hydroxy-2′-deoxyguanosine; AK, Adenylate kinase; AMP, adenosine monophosphate; AMPK, AMP-activated protein kinase; AST, aspartate aminotransferase; ALT, alanine aminotransferase; ATP, adenosine triphosphate; ANOVA, one-way analysis of variance; ARRIVE, Animal Research: Reporting of In Vivo Experiments; CK18, Caspase-cleaved keratin 18; CCL2/MCP-1, Chemokine C-C motif ligand 2/Monocyte Chemoattractant Protein-1; CCL3/MIP-1alpha, CCL3/Macrophage Inflammatory Protein-1alpha; CCL5/RANTES, CCL5/regulated on activation, normal T cell expressed and secreted; CI, cold ischemia; CoQ, Coenzyme Q; COX, cytochrome c oxidase; CXCL1/CINC-1, C-X-C motif chemokine ligand 1/Cytokine-Induced Neutrophil Chemoatractant-1; CXCL5/LIX, CXCL5/Lipopolysaccharide-induced CXC chemokine; CXCL10/IP-10, CXCL10/Interferon-gamma inducible Protein 10kDa; ECD, extended criteria donors; ELISA, enzyme-linked immunosorbent assay; ER, endoplasmic reticulum; ETC, electron transport chain; EVs, extracellular vesicles; FMN, flavin mononucleotide; HPLC, High-performance liquid chromatography; IL, Interleukin; IRI, Ischemia/reperfusion injury; LDH, Lactate dehydrogenase; MSCs, mesenchymal stromal cell; mPTP, mitochondrial permeability transition pore; NMP, Normothermic Machine Perfusion; PK, Pyruvate kinase; PREPARE, Planning Research and Experimental Procedures on Animals: Recommendations for Excellence; RET, reverse electron transport; ROS, reactive oxygen species; SDH, Succinate dehydrogenase; TEM, Transmission electron microscopy; TNF-alpha, Tumor Necrosis Factor-alpha; UPR, unfolded protein response; VEGF, Vascular Endothelial Growth Factor.

## References

[1] Rampes S., Ma D. (2019). Hepatic ischemia-reperfusion injury in liver transplant setting: Mechanisms and protective strategies. J. Biomed. Res..

[2] Dar W.A., Sullivan E., Bynon J.S., Eltzschig H., Ju C. (2019). Ischaemia reperfusion injury in liver transplantation: Cellular and molecular mechanisms. Liver Int..

[3] Xu J., Sayed B.A., Casas-Ferreira A.M., Srinivasan P., Heaton N., Rela M., Ma Y., Fuggle S., Legido-Quigley C., Jassem W. (2016). The impact of ischemia/reperfusion injury on liver allografts from deceased after cardiac death versus deceased after brain death donors. PLoS ONE.

[4] Dutkowski P., Polak W.G., Muiesan P., Schlegel A., Verhoeven C.J., Scalera I., Deoliveira M.L., Kron P., Clavien P.A. (2015). First comparison of hypothermic oxygenated perfusion versus static cold storage of human donation after cardiac death liver transplants. Ann. Surg..

[5] Dondossola D., Ravaioli M., Lonati C., Maroni L., Pini A., Accardo C., Germinario G., Antonelli B., Odaldi F., Zanella A. (2021). The role of ex-situ hypothermic oxygenated machine perfusion and cold preservation time in extended criteria DCD and DBD. Liver Transplant..

[6] Colombo G., Gatti S., Turcatti F., Lonati C., Sordi A., Rossi G., Bonino F., Catania A. (2006). Alteration in the transcriptional profile of livers from brain-dead organ donors. Transplantation.

[7] Soares R.O.S., Losada D.M., Jordani M.C., Évora P., Castro-E-Silva O. (2019). Ischemia/reperfusion injury revisited: An overview of the latest pharmacological strategies. Int. J. Mol. Sci..

[8] Teodoro J.S., Da Silva R.T., Machado I.F., Panisello-Roselló A., Roselló-Catafau J., Rolo A.P., Palmeira C.M. (2022). Shaping of Hepatic Ischemia/Reperfusion Events: The Crucial Role of Mitochondria. Cells.

[9] Panconesi R., Widmer J., Carvalho M.F., Eden J., Dondossola D., Dutkowski P., Schlegel A. (2022). Mitochondria and ischemia reperfusion injury. Curr. Opin. Organ Transplant..

[10] Pell V.R., Chouchani E.T., Murphy M.P., Brookes P.S., Krieg T. (2016). Moving forwards by blocking back-flow the yin and yang of MI therapy. Circ. Res..

[11] Chouchani E.T., Pell V.R., James A.M., Work L.M., Saeb-Parsy K., Frezza C., Krieg T., Murphy M.P. (2016). A unifying mechanism for mitochondrial superoxide production during ischemia-reperfusion injury. Cell Metab..

[12] Bonora M., Giorgi C., Pinton P. (2022). Molecular mechanisms and consequences of mitochondrial permeability transition. Nat. Rev. Mol. Cell Biol..

[13] Chouchani E.T., Pell V.R., Gaude E., Aksentijević D., Sundier S.Y., Robb E.L., Logan A., Nadtochiy S.M., Ord E.N.J., Smith A.C. (2014). Ischaemic accumulation of succinate controls reperfusion injury through mitochondrial ROS. Nature.

[14] Lonati C., Bassani G.A., Brambilla D., Leonardi P., Carlin A., Faversani A., Gatti S., Valenza F. (2018). Influence of ex vivo perfusion on the biomolecular profile of rat lungs. FASEB J..

[15] Roffia V., De Palma A., Lonati C., Di Silvestre D., Rossi R., Mantero M., Gatti S., Dondossola D., Valenza F., Mauri P. (2018). Proteome investigation of rat lungs subjected to Ex vivo perfusion (EVLP). Molecules.

[16] Lonati C., Dondossola D., Zizmare L., Battistin M., Wüst L., Vivona L., Carbonaro M., Zanella A., Gatti S., Schlegel A. (2022). Quantitative Metabolomics of Tissue, Perfusate, and Bile from Rat Livers Subjected to Normothermic Machine Perfusion. Biomedicines.

[17] Guo Z., Zhan L., Gao N., Zhang Z., Huang S., Wang L., Zhu C., Jia Z., Yin M., Li F. (2023). Metabolomics Differences of the Donor Livers Between In Situ and Ex Situ Conditions During Ischemia-free Liver Transplantation. Transplantation.

[18] Lonati C., Schlegel A., Battistin M., Merighi R., Carbonaro M., Dongiovanni P., Leonardi P., Zanella A., Dondossola D. (2021). Effluent Molecular Analysis Guides Liver Graft Allocation to Clinical Hypothermic Oxygenated Machine Perfusion. Biomedicines.

[19] Jassem W., Xystrakis E., Ghnewa Y.G., Yuksel M., Pop O., Martinez-Llordella M., Jabri Y., Huang X., Lozano J.J., Quaglia A. (2019). Normothermic Machine Perfusion (NMP) Inhibits Proinflammatory Responses in the Liver and Promotes Regeneration. Hepatology.

[20] Panconesi R., Flores Carvalho M., Mueller M., Dutkowski P., Muiesan P., Schlegel A. (2021). Mitochondrial Reprogramming—What Is the Benefit of Hypothermic Oxygenated Perfusion in Liver Transplantation?. Transplantology.

[21] Schlegel A., Mergental H., Fondevila C., Porte R.J., Friend P.J., Dutkowski P. (2023). Machine perfusion of the liver and bioengineering. J. Hepatol..

[22] Lonati C., Battistin M., Dondossola D.E., Bassani G.A., Brambilla D., Merighi R., Leonardi P., Carlin A., Meroni M., Zanella A. (2021). NDP-MSH treatment recovers marginal lungs during ex vivo lung perfusion (EVLP). Peptides.

[23] Czigany Z., Tacke F., Lurje I., Lurje G. (2019). Machine perfusion for liver transplantation in the era of marginal organs—New kids on the block. Liver Int..

[24] Miceli V., Bertani A. (2022). Mesenchymal Stromal/Stem Cells and Their Products as a Therapeutic Tool to Advance Lung Transplantation. Cells.

[25] Hoogduijn M.J., Montserrat N., van der Laan L.J.W., Dazzi F., Perico N., Kastrup J., Gilbo N., Ploeg R.J., Roobrouck V., Casiraghi F. (2020). The emergence of regenerative medicine in organ transplantation: 1st European Cell Therapy and Organ Regeneration Section meeting. Transpl. Int..

[26] Grange C., Bellucci L., Bussolati B., Ranghino A. (2020). Potential Applications of Extracellular Vesicles in Solid Organ Transplantation. Cells.

[27] Gatti S., Bruno S., Deregibus M.C., Sordi A., Cantaluppi V., Tetta C., Camussi G. (2011). Microvesicles derived from human adult mesenchymal stem cells protect against ischaemia-reperfusion-induced acute and chronic kidney injury. Nephrol. Dial. Transplant..

[28] Yao J., Zheng J., Cai J., Zeng K., Zhou C., Zhang J., Li S., Li H., Chen L., He L. (2019). Extracellular vesicles derived from human umbilical cord mesenchymal stem cells alleviate rat hepatic ischemia-reperfusion injury by suppressing oxidative stress and neutrophil inflammatory response. FASEB J..

[29] Tian X., Wu L., Li X., Zheng W., Zuo H., Song H. (2024). Exosomes derived from bone marrow mesenchymal stem cells alleviate biliary ischemia reperfusion injury in fatty liver transplantation by inhibiting ferroptosis. Mol. Cell. Biochem..

[30] De Stefano N., Navarro-Tableros V., Roggio D., Calleri A., Rigo F., David E., Gambella A., Bassino D., Amoroso A., Patrono D. (2021). Human liver stem cell-derived extracellular vesicles reduce injury in a model of normothermic machine perfusion of rat livers previously exposed to a prolonged warm ischemia. Transpl. Int..

[31] De Stefano N., Calleri A., Faini A.C., Navarro-Tableros V., Martini S., Deaglio S., Patrono D., Romagnoli R. (2023). Extracellular Vesicles in Liver Transplantation: Current Evidence and Future Challenges. Int. J. Mol. Sci..

[32] Blondeel J., Gilbo N., De Bondt S., Monbaliu D. (2023). Stem cell Derived Extracellular Vesicles to Alleviate ischemia-reperfusion Injury of Transplantable Organs. A Systematic Review. Stem Cell Rev. Rep..

[33] Phinney D.G., Di Giuseppe M., Njah J., Sala E., Shiva S., St Croix C.M., Stolz D.B., Watkins S.C., Di Y.P., Leikauf G.D. (2015). Mesenchymal stem cells use extracellular vesicles to outsource mitophagy and shuttle microRNAs. Nat. Commun..

[34] Peruzzotti-Jametti L., Bernstock J.D., Willis C.M., Manferrari G., Rogall R., Fernandez-Vizarra E., Williamson J.C., Braga A., van den Bosch A., Leonardi T. (2021). Neural stem cells traffic functional mitochondria via extracellular vesicles. PLoS Biol..

[35] Islam M.N., Das S.R., Emin M.T., Wei M., Sun L., Rowlands D.J., Quadri S.K., Bhattacharya S. (2013). Mitochondrial transfer from bone marrow-derived stromal cells to pulmonary alveoli protects against acute lung injury. Nat. Med..

[36] Arslan F., Lai R.C., Smeets M.B., Akeroyd L., Choo A., Aguor E.N.E., Timmers L., van Rijen H.V., Doevendans P.A., Pasterkamp G. (2013). Mesenchymal stem cell-derived exosomes increase ATP levels, decrease oxidative stress and activate PI3K/Akt pathway to enhance myocardial viability and prevent adverse remodeling after myocardial ischemia/reperfusion injury. Stem Cell Res..

[37] Lonati C., Bassani G.A., Brambilla D., Leonardi P., Carlin A., Maggioni M., Zanella A., Dondossola D., Fonsato V., Grange C. (2019). Mesenchymal stem cell-derived extracellular vesicles improve the molecular phenotype of isolated rat lungs during ischemia/reperfusion injury. J. Heart Lung Transplant..

[38] Cillo U., Lonati C., Bertacco A., Magnini L., Battistin M., Consortium L.N.M.P., Borsetto L., Dazzi F., Al-adra D., Gringeri E. (2025). A proof-of-concept study in small and large animal models for coupling liver normothermic machine perfusion with mesenchymal stromal cell bioreactors. Nat. Commun..

[39] Dondossola D., Lonati C., Battistin M., Vivona L., Zanella A., Maggioni M., Valentina V., Zizmare L., Trautwein C., Schlegel A. (2024). Twelve-hour normothermic liver perfusion in a rat model: Characterization of the changes in the ex-situ bio-molecular phenotype and metabolism. Sci. Rep..

[40] Dondossola D., Santini A., Lonati C., Zanella A., Merighi R., Vivona L., Battistin M., Galli A., Biancolilli O., Maggioni M. (2019). Human Red Blood Cells as Oxygen Carriers to Improve Ex-Situ Liver Perfusion in a Rat Model. J. Clin. Med..

[41] Brossa A., Fonsato V., Grange C., Tritta S., Tapparo M., Calvetti R., Cedrino M., Fallo S., Gontero P., Camussi G. (2020). Extracellular vesicles from human liver stem cells inhibit renal cancer stem cell-derived tumor growth in vitro and in vivo. Int. J. Cancer.

[42] Muller X., Schlegel A., Kron P., Eshmuminov D., Würdinger M., Meierhofer D., Clavien P.A., Dutkowski P. (2019). Novel Real-time Prediction of Liver Graft Function During Hypothermic Oxygenated Machine Perfusion Before Liver Transplantation. Ann. Surg..

[43] Monzio Compagnoni G., Kleiner G., Bordoni A., Fortunato F., Ronchi D., Salani S., Guida M., Corti C., Pichler I., Bergamini C. (2018). Mitochondrial dysfunction in fibroblasts of Multiple System Atrophy. Biochim. Biophys. Acta-Mol. Basis Dis..

[44] Meszaros A.T., Hofmann J., Buch M.L., Cardini B., Dunzendorfer-Matt T., Nardin F., Blumer M.J., Fodor M., Hermann M., Zelger B. (2022). Mitochondrial respiration during normothermic liver machine perfusion predicts clinical outcome. eBioMedicine.

[45] Gori F., Fumagalli J., Lonati C., Caccialanza R., Zanella A., Grasselli G. (2022). Ascorbic acid in solid organ transplantation: A literature review. Clin. Nutr..

[46] Saeb-Parsy K., Martin J.L., Summers D.M., Watson C.J.E., Krieg T., Murphy M.P. (2021). Mitochondria as Therapeutic Targets in Transplantation. Trends Mol. Med..

[47] Di Mambro T., Pellielo G., Agyapong E.D., Carinci M., Chianese D., Giorgi C., Morciano G., Patergnani S., Pinton P., Rimessi A. (2023). The Tricky Connection between Extracellular Vesicles and Mitochondria in Inflammatory-Related Diseases. Int. J. Mol. Sci..

[48] Angulski A.B.B., Capriglione L.G., Batista M., Marcon B.H., Senegaglia A.C., Stimamiglio M.A., Correa A. (2017). The Protein Content of Extracellular Vesicles Derived from Expanded Human Umbilical Cord Blood-Derived CD133+ and Human Bone Marrow-Derived Mesenchymal Stem Cells Partially Explains Why both Sources are Advantageous for Regenerative Medicine. Stem Cell Rev. Rep..

[49] Abyadeh M., Amirkhani A., Mirshahvaladi S., Mirzaei M., Kashani S.A., Seydi H., Paulo J.A., Moradpour N. (2024). Proteomic profiling of mesenchymal stem cell-derived extracellular vesicles: Impact of isolation methods on protein cargo. J. Extracell. Biol..

[50] Eden J., Thorne A.M., Bodewes S.B., Patrono D., Roggio D., Breuer E., Lonati C., Dondossola D., Panayotova G., Boteon A.P.C.S. (2024). Assessment of liver graft quality during hypothermic oxygenated perfusion: The first international validation study. J. Hepatol..

[51] Cai J., Chen X., Tang X. (2022). AMPK: The key to ischemia—Reperfusion injury. J. Cell Physiol..

[52] Miceli V. (2026). Priming strategies to enhance the therapeutic efficacy of mesenchymal stromal/stem cell-derived vesicles in regenerative medicine. Extracell. Vesicles Circ. Nucl. Acids.

[53] Calascibetta F., Martorana A., Lo Pinto M., Carcione C., D’Arpa S., Amico G., Miceli V., Cuscino N., Iannolo G., Volpe L. (2025). GMP-compliant, serum-free cultures preserve therapeutic potential of extracellular vesicles from human mesenchymal stromal cells. Front. Cell Dev. Biol..

[54] Calligaris M., Zito G., Busà R., Bulati M., Iannolo G., Gallo A., Carreca A.P., Cuscino N., Castelbuono S., Carcione C. (2024). Proteomic analysis and functional validation reveal distinct therapeutic capabilities related to priming of mesenchymal stromal/stem cells with IFN-γ and hypoxia: Potential implications for their clinical use. Front. Cell Dev. Biol..

