# Supplementary material for: MSC-Derived Extracellular Vesicles Mitigate Ischemia-Induced Energetic Dysfunction During Ex Situ Perfusion of Rat Livers
Source: Antioxidants (Basel). 2026 Jul 4;15(7):843. doi: 10.3390/antiox15070843 (PMC13405841; doi:10.3390/antiox15070843)
Supplement: Supplementary file 1 [file antioxidants-15-00843-s001.zip › antioxidants-4355745-supplementary.pdf]

Supplementary information

ARTICLE TITLE: MSC-derived extracellular vesicles mitigate ischemia-induced energetic dysfunction during ex situ perfusion of rat livers

Table of Contents

S1. Supporting methods ***3***

S1.1. Sample size calculation **3**

S1.2. Anesthesia, surgery, in situ perfusion, and rat liver procurement **3**

S1.3. Rat liver normothermic perfusion **4**

S1.4. Perfusate sample processment **5**

S1.5. Analysis of perfusate samples **5**

S1.5.1. Succinate 5

S1.5.2. Flavin mononucleotide (FMN) 5

S1.5.3. 8-hydroxy-2'-deoxyguanosine (8-OHdG) 5

S1.5.4. Cell death markers: Caspase-cleaved keratin 18 (CK18) and Adenylate kinase (AK) 6

S1.5.5. Ketone bodies 6

S1.5.6. Molecules relevant to inflammation and its resolution 6

S1.5.7. Human IL-4, IL-6, IL-10, IL-13, IL-1ra, IL-36-beta, Galectin-3 and CCL-2/MCP-1 7

S1.6. Evaluations performed on rat liver biopsies **7**

S1.6.1. Wet-to-Dry Ratio 7

S1.6.2. Histological and histochemical analysis 7

S1.6.3. Ultrastructural analysis by Transmission electron microscopy (TEM) 7

S1.6.4. Activity of glycolysis enzymes and ETC complexes 8

S1.6.5. ATP content assessment 8

S1.6.6. ATP derivatives by High-performance liquid chromatography (HPLC) 8

S1.6.7. Western blot to assess AMP-activated protein kinase (AMPK) signaling 8

S2. Supplementary figures ***10***

Supplementary figure S1. Bile output **10**

Supplementary figure S2. Morphologyical evaluation a of liver biopsies 11

Supplementary figure S3. Activity of Lactate dehydrogenase (LDH) 12

Supplementary figure S4. Immunohistochemistry of liver biopsies 13

Supplementary figure S5. Activity of Succinate dehydrogenase (SDH) 14

Supplementary figure S6. Activity of mitochondrial Complex II+III 15

Supplementary figure S7. Activity of NADH dehydrogenase 16

Supplementary figure S8. Activity of Pyruvate kinase 17

Supplementary figure S9. Inosine content in liver tissue homogenates 18

Supplementary figure S10. Perfusate concentration of transaminases and LDH 19

Supplementary figure S11. Perfusate concentration of K+ during the NMP procedure 20

Supplementary figure S12. Perfusate concentration of mediators of human origin 21

S3. Supplementary tables *22*

Supplementary table S1. Hemodynamics monitoring during NMP 22

Supplementary table S2. Gas-analysis of perfusate samples 23

Supplementary table S3. Bile gas-analysis 26

S4. References *28*

S1. Supporting methods

S1.1. Sample size calculation

The experiments conducted in the present research were planned according to the Planning Research and Experimental Procedures on Animals: Recommendations for Excellence (PREPARE) guidelines (1) and performed in compliance with the 3R principles (2,3).

An "a priori" power analysis was carried out sing SigmaStat software 11.0 (Systat Software Inc, San Jose, CA, USA) to estimate the minimum number of rats needed to reliably detect the expected effect size (1–3). Given the well-known negative effects of reperfusion on mitochondrial oxidative phosphorylation, ATP liver content was used as reference variable to explore the effects exerted by the bioreactor-based perfusion. Details on sample size calculation are listed below:

- number of groups: 5 (Native, NMP, NMP+EVs, CI+NMP, CI+NMP+EVs);
- statistical test: one-way or two-way repeated measures ANOVA;
- expected effect size: 0.45;
- expected standard deviation of residuals: 0.20;
- desired power: 0.80;
- alpha error: 0.05.

The analysis provided a minimum number of 5 livers for each experimental group. The final number needed to perform the whole study was therefore 25 rat livers.

S1.2. Anesthesia, surgery, in situ perfusion, and rat liver procurement

All experiments were performed in sterile conditions. Rats were anesthetized using Isoflurane (Iso-Vet, Piramal Critical Care, Voorschoten, Netherlands) 3-4% (1-2 L/min O_2 flow) in a rat anesthetic induction chamber for 10 min. When unresponsive to pain, rats were placed onto the operating table, ensuring spontaneous breathing with a mask that insufflate 1 L/min O_2 and Isoflurane 3-4%.

The surgical procedure began with a xifo-pubic and bilateral subcostal incision. The hepatic pedicle was then exposed, and the bile duct was dissected. Bile duct was distally ligated with an 8-0 nylon tie, then a customized cannula (tip: 24G Neo Delta Ven, Delta Med SpA, Viadana, Italy; tube: PE-50, BD Intramedic™, Franklin Lakes, NJ, US) was inserted at the end of the choledotomy; the cannula was secured with a proximal 8-0 nylon tie. Then, the pyloric vein was ligated (8-0 nylon) and transected, and then the main trunk of the portal vein was dissected distally. The portal vein was encircled with three 5-0 silk tie; then unfractioned heparin (2 IU/g diluted in 1 ml of saline, Epsoclar,

Pfizer Italia S.r.l., Latina, Italy) was administered by tail intravenous injection. After three min, portal vein was ligated distally to the splenic vein insertion and a 16 G cannula (Neo Delta Ven, Delta Med SpA) was inserted by venipuncture until the tip of the cannula reached the portal bifurcation. A blood retrograde flush was performed, and the in-situ perfusion system was connected. Then, the cannula was secured with a two-silk tie and gently retracted to avoid obstruction of the right lobe portal vein branch. Rapid sternotomy was performed, the heart was removed, and the inferior vena cava was transected. In-situ cold perfusion (4°C) was started with 35 ml of Celsior solution (IGL, Lissieu, France) at a pressure of 30 cmH_2O. At the end of in-situ cold perfusion, hepatectomy was completed and the liver graft was stored in 4°C Celsior (IGL) solution for 30 min. At the end of cold storage, backtable was completed, while maintaining the liver on a refrigerated surface.

S1.3. Rat liver normothermic perfusion

At the end of static cold storage (SCS), the liver was placed onto the modified heated glass reservoir to let the liver laid on the diaphragmatic surface on a perforated Parafilm. The liver was then connected to the circuit through the portal vein cannula, while the bile duct cannula was connected to a 1.5 ml tube in order to collect bile during reperfusion. The chamber was closed to maintain humidity. We carefully avoided air embolism during priming and liver connection. The membrane oxygenator was ventilated with 50% FiO2 and 5% $FiCO_2$, 200 ml/min gas flow.

Liver perfusion was performed according to the protocol previously reported by our research group (4), using a customized circuit derived from an isolated lung perfusion system (Hugo Sachs Elektronik, Harvard Apparatus, March-Hugstetten, Germany).

The NMP-protocol was maintained for 280 min and included two phases: 1) the rewarming phase, lasting 40 min and 2) the normothermic phase, lasting 240 min. During the “rewarming” phase, the system reaches the temperature of 37°C. Portal flow was set at 5 ml/min and was increased every 5 min up to 30 ml/min, or until the portal pressure reaches 8 mmHg. In the normothermic phase, temperature was maintained at 37°C and portal flow remained unchanged.

The perfusion solution used in the present research was prepared under sterile conditions as previously described (4) and supplemented with an oxygen carrier (Oxyglobin®, HBO2 Therapeutics, Boston, USA). Perfusion fluid volume was maintained at 100 ml throughout the procedure. To prevent MethOxyglobin accumulation, from the second hour of normothermic phase a fixed volume of perfusate was replaced with fresh perfusion fluid at 20 ml/h flow rate. Waste flow rate was 8 ml/h from 1 h to 2 h, while it was increased to 12 ml/h from 2 h to 4 h to adjust for the volume of perfusate collected for biomolecular analysis.

The perfusion protocol involved continuous pressure and temperature monitoring with hourly evaluation of perfusate acid-base balance, electrolytes, and metabolite concentration (ABL 800 Flex, Radiometer Medical ApS, Copenhagen, Denmark). Hemodynamics parameters were monitored using the Powerlab 16/35 system (ADInstruments, Dunedin, New Zeland) and LabChart 8 pro (ADInstruments).

S1.4. Perfusate sample processment

Perfusate samples were centrifugated at 2000 rpm (700 g) for 10 min at 4°C (Heraeus Multifuge X3R, Thermo Fisher Scientific, Waltham, MA, USA). Supernatants were collected and purified with Amicon Ultra 100 kDa filters (Millipore Corporation, Merck KGaA, Darmstadt, Germany) to avoid colorimetric interferences possibly given by Oxyglobin®.

S1.5. Analysis of perfusate samples

After perfusate processing as reported in the previous paragraph, samples were used to assess the following parameters:

S1.5.1. Succinate

Perfusate succinate was determined by a coupled enzyme reaction, which results in a colorimetric product proportional to the succinate present (Succinate Colorimetric Assay Kit, Sigma-Aldrich, Merck KGaA, Darmstadt, Germany). Perfusates were deproteinized using Amicon Ultra 10K centrifugal filter devices (Millipore Corporation). Standards were prepared adding from 10 µL to 2 µL of 1 mM standard solution, producing a concentration scale ranging from 10 nmol/well to 2 nmol/well. After a 30-min incubation at 37°C, absorbance was read at 450 nm (Synergy HTX, Biotek U.S, Winooski, VT, USA).

S1.5.2. Flavin mononucleotide (FMN)

FMN concentration was determined by fluorescence spectroscopy according to previously published protocols (5,6). Briefly, perfusate samples were dispensed in triplicate in black 96-well microplates (Promega, Madison, WI, USA). Fluorescence reading was performed with an excitation wavelength of 460/40 nm, while fluorescence emission was detected with 100% gain at 528/20 nm (Synergy HTX).

S1.5.3. 8-hydroxy-2'-deoxyguanosine (8-OHdG)

The release of oxidized form of 2’-deoxyguanosine was assessed as a biomarker of oxidative damage to DNA (DNA damage competitive ELISA, Thermo Fisher Scientific). Absorbance reading was performed at 450 nm using a multi-mode microplate reader (Synergy HTX).

S1.5.4. Cell death markers: Caspase-cleaved keratin 18 (CK18) and Adenylate kinase (AK)

Liver cell apoptosis was assessed by measuring the release of caspase-cleaved CK18 with a commercially available immunoassay based on the use of M30 monoclonal antibody that specifically detects the neoepitope ccK18/K18-Asp396 (Cusabio Technology LLC, Houston, USA). Absorbance readings were carried out using a multi-mode microplate reader (Synergy HTX).

Aspecific cell death was evaluated by measuring the release of adenylate kinase (AK) from damaged cells using the ToxiLight™ bioassay kit (Lonza Bioscience, Basel, Switzerland). Bioluminescent signals were then acquired using a Luminometer (Promega).

S1.5.5. Ketone bodies

Liver production of 3-hydroxybutyric acid (BOH) and acetoacetic acid (AcAc) was determined by measuring their concentration in perfusate samples using an enzymatic assay based on 3-hydroxybutyrate dehydrogenase catalyzed reactions (Ketone Body Assay Kit, Sigma-Aldrich). Absorbance readings at 340 nm were carried out using a multi-mode microplate reader (Synergy HTX).

S1.5.6. Molecules relevant to inflammation and its resolution

Soluble proteins relevant to immune activation were evaluated by means of Luminex® xMAP Technology (Luminex 200; Luminex, Austin, TX, USA) with a custom-designed Rat Cytokine/Chemokine Magnetic Bead Panel (EMD Millipore Corporation, Billerica, MA, USA), including the following molecules: Chemokine C-C motif ligand 2/Monocyte Chemoattractant Protein-1 (CCL2/MCP-1); Chemokine C-C motif ligand 3/Macrophage Inflammatory Protein-1alpha (CCL3/MIP-1alpha); Chemokine C-C motif ligand 5/regulated on activation, normal T cell expressed and secreted (CCL5/RANTES); C-X-C motif chemokine ligand 1/Cytokine-Induced Neutrophil Chemoatractant-1 (CXCL1/CINC-1); C-X-C Motif Chemokine Ligand 5/Lipopolysaccharide-induced CXC chemokine (CXCL5/LIX); C-X-C Motif Chemokine Ligand 10/Interferon-gamma inducible Protein 10kDa (CXCL10/IP-10); Interleukin-4 (IL-4); Interleukin-6 (IL-6); Interleukin-10 (IL-10); Interleukin-18 (IL-18); Vascular Endothelial Growth Factor (VEGF); Tumor Necrosis Factor-alpha (TNF-alpha).

Briefly, 150 µl from each antibody-bead vial were pooled in a mixing bottle and then brought to a final volume of 3 ml with the kit-specific Assay Buffer. Twenty-five µl of standards, controls, and samples were dispensed to the appropriate wells, then each well was brought to a final volume of 75 µl with 25 µl of Assay Buffer and 25µl of Mixed Beads. The plate was incubated in the dark for 2 h with gentle agitation at RT. Plate was washed 3 times, then Detection Antibodies and Streptavidin-

Phycoerythrin were added to the wells. Lastly, 150 µl of Sheath Fluid were added for subsequent fluorescence detection using a Luminex 200 (Luminex).

S1.5.7. Human IL-4, IL-6, IL-10, IL-13, IL-1ra, IL-36-beta, Galectin-3 and CCL-2/MCP-1

The concentration of Human IL-4, IL-6, IL-10, IL-13, IL-1ra, IL-36-beta, Galectin-3 and CCL-2/MCP-1 was measured by means Luminex® xMAP Technology (Luminex 200) with a custom-designed immunoassays (R&D Systems, Minneapolis, MN, USA). Briefly, 100 µl from each antibody-bead vial were added to a Mixing Bottle and then brought to a final volume of 1 ml with the kit-specific Assay Buffer. Fifty µl of standards, controls and samples were added to the appropriate wells, then each well was brought to a final volume of 100 µL with 50 µl of mixed beads. The plate was incubated in the dark for 2 h with gentle agitation at RT. Plate was washed 3 times for every new step; then Biotin and Streptavidin were added subsequently to the wells. Lastly, 100 µl of Wash Buffer were added for fluorescence detection.

S1.6. Evaluations performed on rat liver biopsies

S1.6.1. Wet-to-Dry Ratio

Biopsies were weighted on an analytical balance (Sartorius AG, Göttingen, Germany) before and after desiccation in an oven at 50 °C for 48 h. Thereafter, wet/dry ratios (W/D) were calculated as an index of edema.

S1.6.2. Histological and histochemical analysis

Liver biopsies frozen in isopentane pre-cooled with liquid nitrogen were processed as previously described (7) to obtain 8 µm-thick cryosections. Next, staining with Hematoxylin and Eosin was performed for standard morphological evaluation, while immunohistochemistry was carried out to evaluate the presence and abundance of cytochrome c oxidase (COX) and succinate dehydrogenase (SDH) (Sigma-Aldrich).

S1.6.3. Ultrastractural analysis by Transmission electron microscopy (TEM)

After overnight incubation in 2.5% glutaraldehyde, liver biopsies were postfixed in 2% osmium tetroxide (OsO_4) (Sigma-Aldrich) for 1 h. Thereafter, tissue specimens were dehydrated with increasing ethanol series (from 60% to 100%), embedded in an Epon resin (EMS) and polymerized in an oven (ISCO) at 62°C for 48 h. Ultrathin (70–90 nm) sections were collected on nickel grids, stained with uranyl acetate replacement (UAR), lead citrate and observed with a Transmission Electron Microscope (Hitachi HT7800, Japan).

S1.6.4. Activity of glycolysis enzymes and ETC complexes

The activities of glycolytic enzymes and of enzymes of each respiratory chain complex were measured in tissue homogenates as previously described (8–10). Analyses were performed using a Parkin Elmer software. Measurements were normalized over the activity level of citrate synthase, a stable matrix mitochondrial enzyme; this latter step was performed in order to normalize respiratory chain activity over mitochondrial mass.

S1.6.5. ATP content assessment

Snap frozen liver samples were homogenized in trichloroacetic acid (Sigma-Aldrich) at a concentration of 50 mg/ml. Liver homogenates were subjected to centrifugation at 20817 g for 15 min at 4°C (Heraeus Multifuge X3R) and then supernatants were collected and diluted 1:30 using 0.1 M Tris-acetate, pH 7.75 (Millipore, Merck KGaA). Next, 10 µl of each sample were dispensed in a blank 96-well plate and 90 µL of luciferase (Enliten ATP Assay System, Promega, Madison, WI, USA) were automatically added during the analysis in the Luminometer (Glomax Luminometer, Promega). Bioluminescent signals were immediately detected. ATP concentration was calculated by means of a standard curve that ranged from 10^{-4} M to 10^{-10} M (rATP 10 mM, Promega).

S1.6.6. ATP derivatives by High-performance liquid chromatography (HPLC)

Snap frozen tissue biopsies (40-50 mg) were homogenized in 2.5% trichloroacetic acid (Sigma-Aldrich) at a concentration of 50 mg/ml. Liver homogenates were subjected to centrifugation at 20817 g for 15 min at 4°C (Heraeus Multifuge X3R) and then supernatants were analyzed in duplicates by High-performance liquid chromatography (HPLC) with a mass spectrometer as detector (UltiMate 3000, Thermo Fisher Scientific) using a reversed phase C18 HPLC column (Zorbax RRHD SB-C18, 5micron, 4.6x250 mm, Agilent, Santa Clara, CA, US), with a mobile phase containing 0.1% formic acid in water (fase A) and CH3CN (fase B). The signal was detected by UV absorbance at 254 nm. Energy charge defined as: (ATP + 0.5 x ADP) / (ATP + ADP + AMP) was calculated as previously described (11).

S1.6.7. Western blot to assess AMP-activated protein kinase (AMPK) signaling

Snap frozen liver biopsies were homogenized in an appropriate volume of T-PER™ Tissue Protein Extraction Reagent (Thermo Fisher Scientific) added with phosphatase and protease inhibitors (Sigma-Aldrich). Tissue lysates were then centrifuged at 14.000 rpm (20,000 g) at 4°C for 30 min (Heraeus Multifuge X3R). Supernatants were collected and used to determine protein concentration by a bicinchoninic acid-based method (Bio-Rad, Hercules, CA, US). Forty micrograms of proteins were subjected to SDS-polyacrylamide gel electrophoresis using mPAGE™ 4-20% Bis-Tris Precast

gels (Merck Millipore) on a Bio-Rad electrophoresis system. Fractionated proteins were transferred onto PVDF membranes and blocked for 1 h at room temperature (RT) with 5% BSA (Sigma–Aldrich). Subsequently, membranes were incubated overnight at 4°C with primary rabbit antibodies against either Phospho-AMPKα (Thr172) (1:2,000, #2531 Cell Signaling Technologies, Denver, MA, US) or AMPKα (1:1,000, #2532 Cell Signaling Technologies). To identify correctly the specific bands, AMPK Control Cell Extracts (BK9158S, Cell Signaling Technologies) were also used. After multiple washing with TBST buffer (Bio-Rad), blots were incubated with a HRP-conjugated secondary antibody (1:2,000 Santa Cruz Biotechnology, Santa Cruz, CA) for 1 h at RT. Membranes were developed using SuperSignal™ West Pico PLUS Chemiluminescent Substrate (Thermo Fisher Scientific) reagents following the manufacturer's instructions. Luminescent signals were detected with an imaging system (Molecular Imager ChemiDoc XRS, Bio Rad). Densitometric analysis was performed using Chemidoc imaging system software (Bio-Rad). Thereafter, membranes were subjected to band stripping to remove primary and secondary antibodies (Bio-Rad) and re-probed using a rabbit monoclonal anti GAPDH antibody (1:2,000; Santa Cruz Biotechnology), followed by incubation with HRP-conjugated secondary antibody (1:5,000; Santa Cruz Biotechnology).

S2. Supplementary figures

Supplementary figure S1. Bile output

One-way ANOVA, Tukey's post hoc test

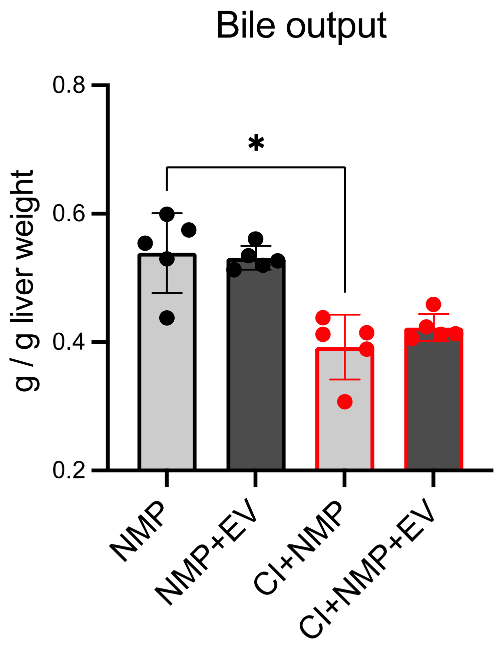

Supplementary figure S2. Morphologyical evaluation a of liver biopsies

Representative H&E images of the different experimental groups: A) Native; B) CI+NMP; C) CI+NMP+EV showing normal morphology in all examined tissues.

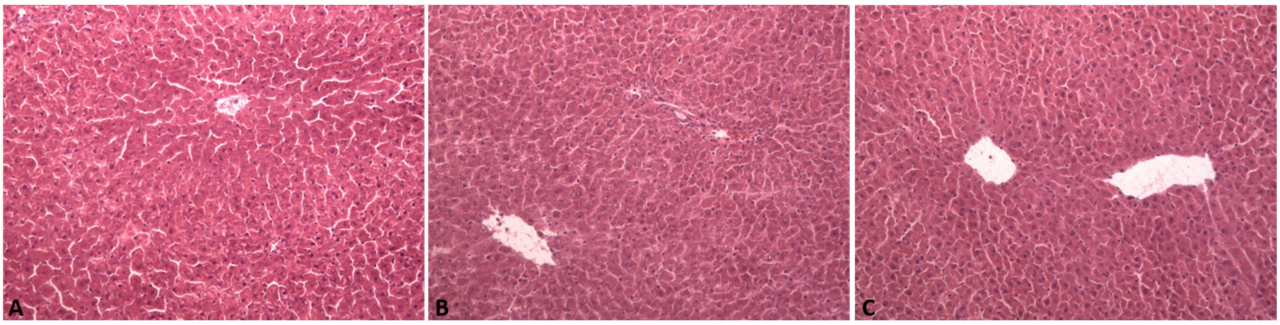

Supplementary figure S3. Activity of Lactate dehydrogenase (LDH)

Kruskall-Wallis test, followed by Dunn's test.

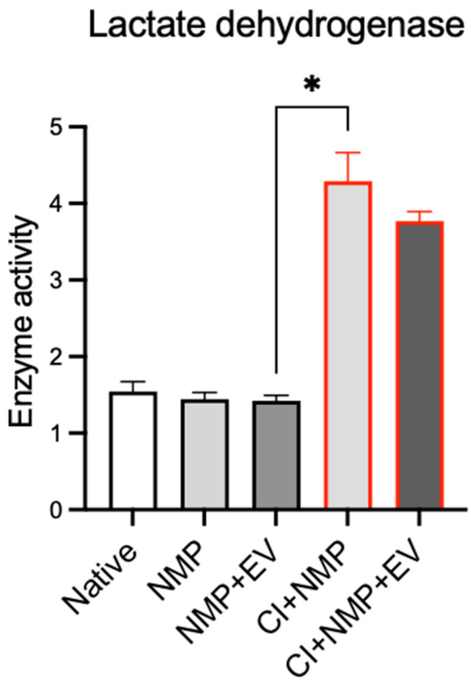

Supplementary figure S4. Immunohistochemistry of liver biopsies

Immunohistochemistry revealed similar staining for COX in livers exposed to CI: CI+NMP (B), CI+NMP+EV (C) compared to the Native group (A). Scale Bar: 50 µm.

A) B) C)

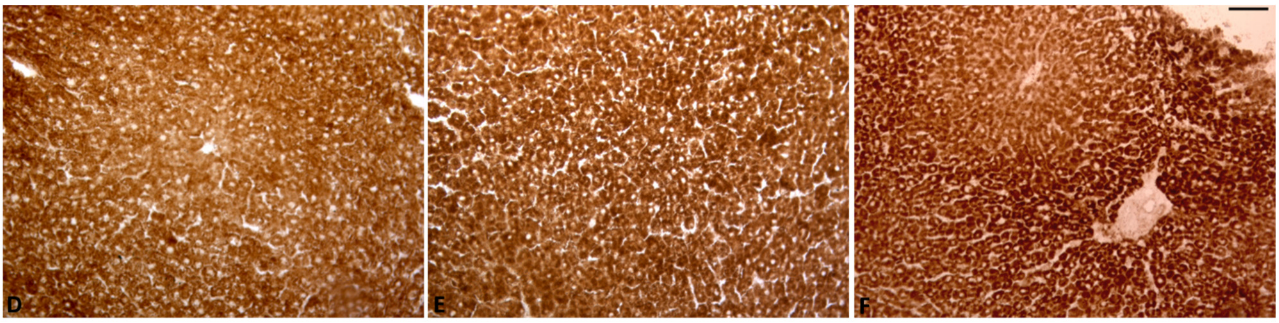

Supplementary figure S5. Activity of Succinate dehydrogenase (SDH)

Kruskall-Wallis test, followed by Dunn's test.

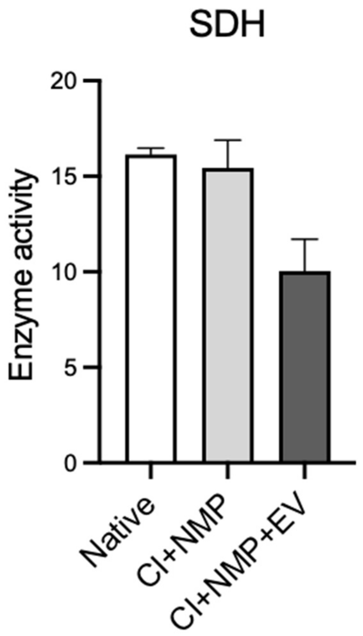

Supplementary figure S6. Activity of mitochondrial Complex II+III

Kruskall-Wallis test, followed by Dunn’s test.

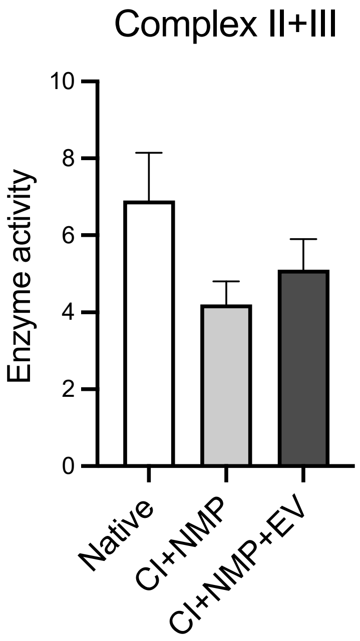

Supplementary figure S7. Activity of NADH dehydrogenase

Kruskall-Wallis test, followed by Dunn's test.

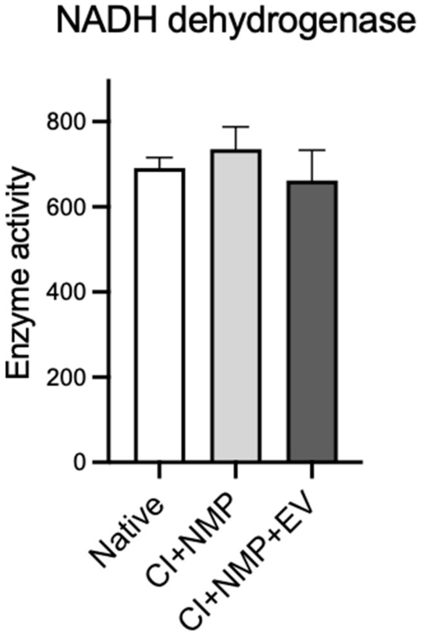

Supplementary figure S8. Activity of Pyruvate kinase

Kruskall-Wallis test, followed by Dunn’s test.

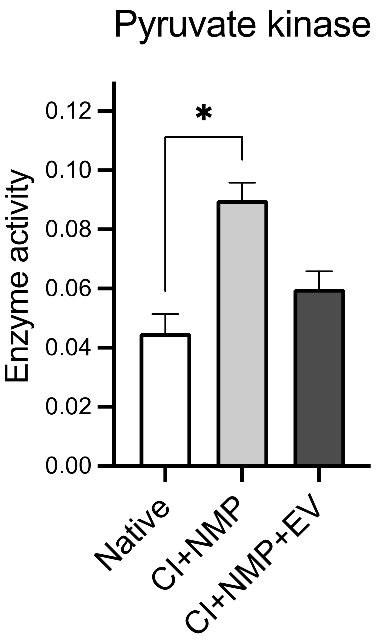

Supplementary figure S9. Inosine content in liver tissue homogenates

One-way ANOVA; Tukey's post hoc test.

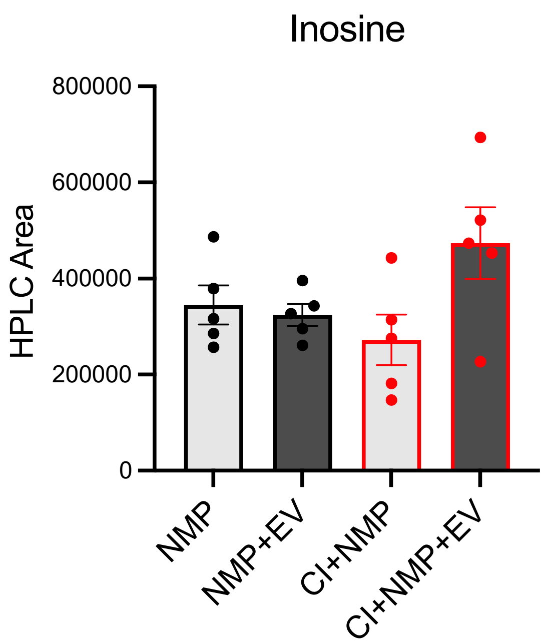

Supplementary figure S10. Perfusate concentration of transaminases and LDH

Two-way RM ANOVA, Tukey's post hoc test

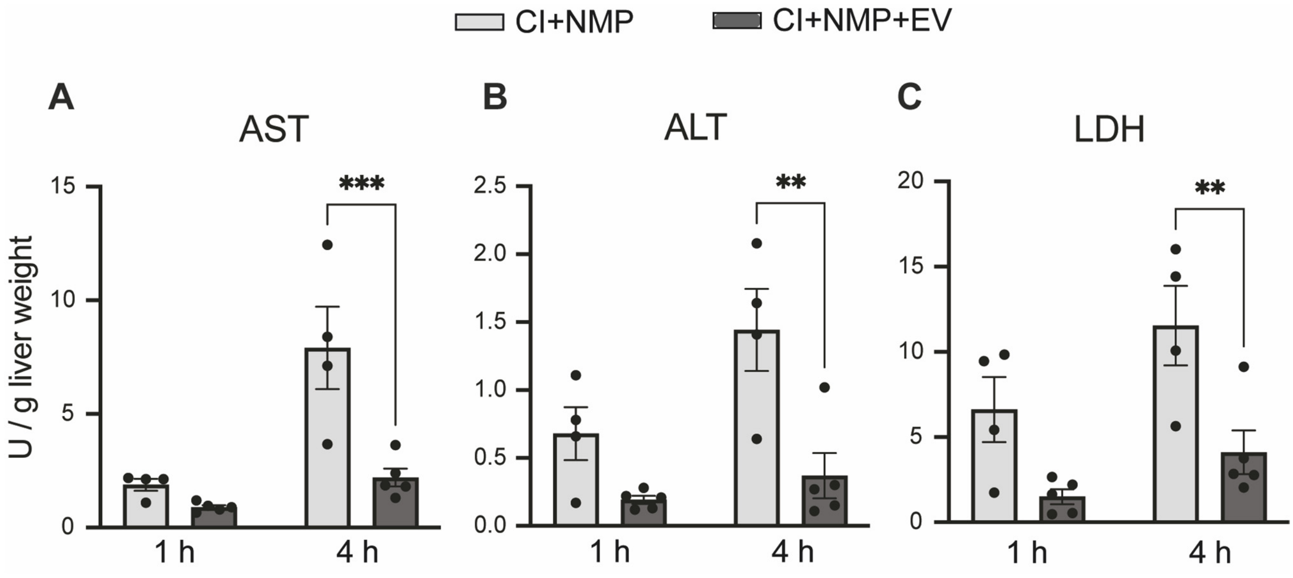

Supplementary figure S11. Perfusate concentration of K+ during the NMP procedure

Two-way RM ANOVA, Tukey's post hoc test

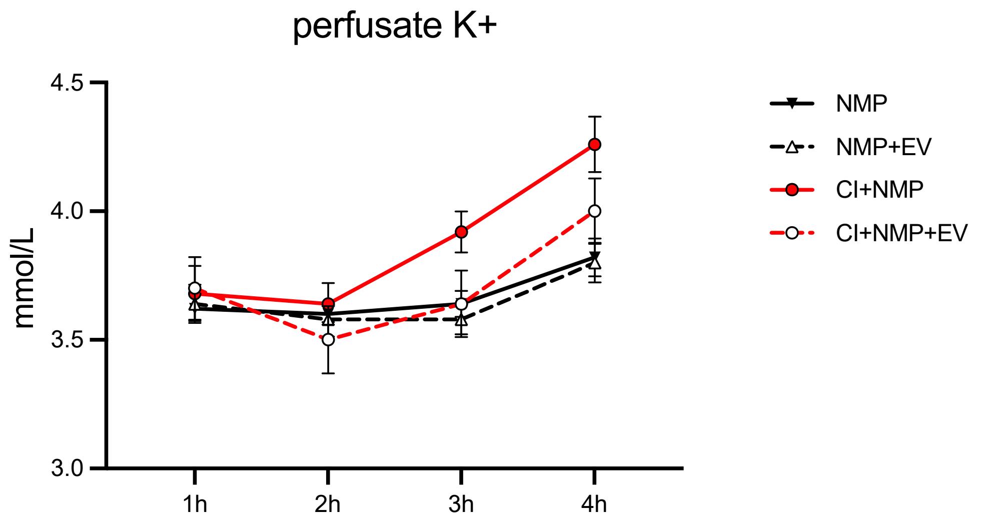

Supplementary figure S12. Perfusate concentration of mediators of human origin

Changes in the levels of Galectin-3, IL-10, and IL-1ra suggest liver uptake of these EV-delivered mediators. Two-way RM ANOVA, Tukey's post hoc test; p-values were FDR-adjusted. Abbreviations: CI, cold ischemia; EVs, extracellular vesicles; FC, Fold change; NMP, normothermic machine perfusion.

		Total amount			
**Human Mediator**	**CI**	0h	1h	4h	**p-value**
**IL-6**	-	81	88	75	0.577
	+	77	84	71	
**Galectin-3**	-	2691	2222	2822	**0.034**
	+	1366	1200	1951	
**IL-10**	-	239	568	566	**0.012**
	+	136	314	360	
**IL-1ra**	-	5647	3346	3647	**0.006**
	+	2348	2821	2522	
**IL-4**	-	247	330	324	0.136
	+	139	137	303	

Perfusate picograms

MIN		MAX
71	2500	5467

S3. Supplementary tables

Supplementary table S1. Hemodynamics monitoring during NMP

Two-way RM ANOVA, Tukey’s post hoc test

						p-value	
**Variable**	**Time**	**NMP**	**NMP+EV**	**CI+NMP**	**CI+NMP+EV**	**all groups**	**CI+NMP+EV vs CI+NMP**
**Portal pressure (mmHg)**							
	1h	7.04±0.42	7.04±0.22	7±0.19	7.2±0.16		
	2h	7.44±0.45	7.32±0.2	6.96±0.14	7.26±0.22	ns	ns
	3h	7.66±0.45	7.46±0.26	7.42±0.13	7.51±0.28		
	4h	7.62±0.42	7.62±0.31	7.82±0.18	7.86±0.23		
**Portal resistances (mmHg min/ml)**							
	1h	0.23±0.01	0.23±0	0.23±0	0.24±0		
	2h	0.24±0.01	0.24±0	0.23±0	0.24±0	ns	ns
	3h	0.25±0.01	0.24±0	0.24±0	0.25±0		
	4h	0.25±0.01	0.25±0.01	0.26±0	0.26±0		

Supplementary table S2. Gas-analysis of perfusate samples

Two-way RM ANOVA, Tukey's post hoc test

						p-value	
**Variable**	**Time**	**NMP**	**NMP+EV**	**CI+NMP**	**CI+NMP+EV**	**all groups**	**CI+NMP+EV vs CI+NMP**
**pH**							
	1h	6.97±0.02	6.96±0.01	6.95±0	6.94±0.01		
	2h	7.06±0	7.05±0	7.01±0.01	7.03±0	<0.001	ns
	3h	7.07±0	7.06±0	7.01±0.01	7.05±0		
	4h	7.06±0	7.05±0	6.99±0.01	7.02±0		
**pCO2 (mmHg)**							
	1h	45.18±0.53	44.3±0.7	48.4±1.22	46.42±0.88		
	2h	46.32±0.49	46±0.51	49.16±1.04	46.18±0.92	0.001	**<0.05**
	3h	46.18±0.58	46.53±0.65	49.24±0.84	46.22±0.84		
	4h	46.06±0.51	46.63±0.71	49.56±0.59	46.18±0.97		
**pO2 (mmHg)**							
	1h	31.6±1.26	34.9±0.94	27.24±3.17	28.34±3.81		
	2h	45.44±0.75	44±2.19	37.52±2.89	40.5±4.03	<0.001	**<0.05**
	3h	52.9±2.03	52.01±2.66	42.86±6.58	54.68±6.9		

4h	59.9±4.61	59.11±4.91	40.2±4.45	57.74±5.75		
**K+ (mmol/L)**						
1h	3.62±0.02	3.65±0.06	3.68±0.1	3.7±0.12		
2h	3.6±0.03	3.56±0.05	3.64±0.08	3.5±0.13	0.007	**<0.05**
3h	3.64±0.05	3.61±0.06	3.92±0.08	3.64±0.12		
4h	3.82±0.07	3.86±0.09	4.26±0.1	4±0.12		
**Na+ (mmol/L)**						
1h	157.2±1.31	158.16±1.04	155.4±0.5	159.8±2.69		
2h	159.6±1.93	158±1.46	157.6±0.67	160±2.64	0.400	ns
3h	159±0.7	156.33±0.8	159.2±0.58	158.2±3.26		
4h	160.4±1.28	158.5±1.17	158.6±0.4	161.8±2.15		
**Ca++ (mmol/L)**						
1h	0.93±0.01	0.96±0	0.89±0	0.91±0.03		
2h	0.93±0.01	0.92±0.01	0.87±0	0.87±0.03	<0.001	ns
3h	0.91±0.01	0.88±0.01	0.88±0	0.83±0.02		
4h	0.91±0	0.9±0.01	0.87±0	0.85±0.01		
**Cl- (mmol/L)**						
1h	116±0.7	114.16±0.3	116.6±0.67	115.4±2.89		
2h	116.6±0.87	116.33±0.71	117.8±0.58	120.4±2.46	<0.001	**<0.05**
3h	117.8±0.58	117±0.89	119±0.63	127.4±3.32		

4h	117.6±0.5	117.33±1.05	119.4±0.5	126.2±1.35		
**Glu (mg/dL)**						
1h	220.2±20.08	223.83±10.59	251.2±7.69	226.6±9.1		
2h	164±10.21	184.5±8.12	193±8.97	175.8±9.33	0.004	**<0.05**
3h	204±16.26	215±10.33	210.6±10.86	173.4±11.99		
4h	220.4±12.38	252.33±9.3	247.8±11.94	208±19.74		
**Lac (mmol/L)**						
1h	1.7±0.45	1.75±0.36	2.85±0.3	3.12±0.1		
2h	1.02±0.15	1.01±0.09	2.35±0.35	2.68±0.12	<0.001	ns
3h	1.52±0.13	1.6±0.1	2.64±0.31	2.76±0.09		
4h	1.92±0.19	2.08±0.12	3.02±0.28	3.1±0.08		
**cBase(Ecf) (mmol/L)**						
1h	-19.4±0.63	-19.85±0.67	-19.32±0.29	-20.02±0.71		
2h	-15.82±0.36	-15.95±0.27	-16.86±0.38	-16.98±0.36	<0.001	ns
3h	-15.4±0.24	-15.4±0.19	-17.02±0.38	-16.24±0.42		
4h	-15.84±0.31	-15.85±0.23	-17.56±0.45	-17.34±0.54		

Supplementary table S3. Bile gas-analysis

Two-way RM ANOVA, Tukey's post hoc test

						p-value	
**Variable**	**Time**	**NMP**	**NMP+EV**	**CI+NMP**	**CI+NMP+EV**	**all groups**	**CI+NMP+EV vs CI+NMP**
**pH**							
	1h	7.04±0.01	7.05±0.01	7.02±0.02	7.03±0.01	0.002	ns
	2h	7.21±0.01	7.2±0.01	7.18±0.01	7.18±0.01		
	3h	7.24±0	7.25±0.01	7.2±0	7.22±0		
	4h	7.24±0.01	7.23±0.01	7.18±0.02	7.21±0		
**pCO2 (mmHg)**							
	1h	35.92±0.37	35.51±0.69	35.04±0.61	35.24±1.04	ns	ns
	2h	39.2±0.43	38.91±0.82	40.38±1.04	38.82±0.97		
	3h	40.9±0.43	40.1±0.75	41.04±0.76	40.32±0.71		
	4h	41.34±0.24	41.6±0.56	42.9±2.5	40.64±0.59		
**pO2 (mmHg)**							
	1h	6.17±2.23	6.23±1.86	3.46±0.25	3.18±1.75	<0.001	ns
	2h	6.5±1.67	9.93±1.75	2.7±0.6	3.46±1.18		
	3h	8.48±1.55	12.66±1.59	3.36±0.82	2.9±0.87		
	4h	8.46±1.34	9.31±1.94	4.6±1.19	3.07±1.45		
**K+ (mmol/L)**							
	1h	3.5±0.08	3.55±0.1	3.9±0.07	3.58±0.14	<0.001	**<0.05**
	2h	4.16±0.04	4.1±0.05	4.18±0.05	3.98±0.12		
	3h	4.1±0.08	4.08±0.07	4.34±0.06	3.94±0.14		
	4h	4.34±0.09	4.26±0.1	4.82±0.06	4.34±0.16		
**Na+ (mmol/L)**							
	1h	159.2±1.85	160.16±1.1	161±1.3	161.8±2.95	ns	ns
	2h	159.8±2.13	159±1.46	157±1.14	159.4±3.1		

3h	159.2±0.86	157.33±0.84	158.2±1.06	157.2±3.27		
4h	160.6±1.43	159.83±1.13	160.2±0.37	162.2±2.26		
**Ca++ (mmol/L)**						
1h	0.76±0.02	0.82±0.01	0.72±0.02	0.72±0.04		
2h	0.77±0.02	0.79±0.02	0.7±0	0.69±0.03	<0.001	ns
3h	0.76±0.01	0.75±0.01	0.7±0	0.66±0.02		
4h	0.76±0.01	0.78±0.01	0.72±0.01	0.71±0.01		
**Cl- (mmol/L)**						
1h	105.4±0.92	105.33±0.61	99.4±0.81	108±3.3		
2h	109.6±0.81	109.5±0.92	106.6±0.81	113.2±2.81	<0.001	**<0.05**
3h	111.8±0.37	111.5±1.05	109.8±0.86	121±2.98		
4h	112.2±0.37	112.33±1.17	110.4±0.97	121±1.22		
**Glu (mg/dL)**						
1h	46.6±16.16	72.16±10.41	43.2±4.27	46.6±7.55		
2h	22.25±6.01	39.83±5.23	33.4±6	25.4±6.06	0.001	ns
3h	44.4±16.01	33.16±5.24	21.8±5.72	15.2±3.73		
4h	41.8±7.43	65.83±8.73	40.8±8.64	28±7.94		
**Lac (mmol/L)**						
1h	3.54±0.18	3.28±0.3	4.26±0.38	3.48±0.17		
2h	1.26±0.29	1.33±0.14	2.16±0.33	2.64±0.05	<0.001	ns
3h	1.3±0.15	1.35±0.07	2.22±0.31	2.5±0.07		
4h	1.84±0.13	2.01±0.13	2.84±0.28	2.88±0.09		
**HCO3- (mmol/L)**						
1h	9.36±0.47	9.55±0.45	8.78±0.38	8.96±0.46		
2h	15.18±0.57	14.65±0.49	14.6±0.27	14.18±0.52	<0.001	ns
3h	17.1±0.23	16.98±0.37	15.82±0.34	16.2±0.41		
4h	17.38±0.39	17.08±0.41	15.3±0.44	15.68±0.45		

S4. References

1. Smith AJ, Clutton RE, Lilley E, Hansen KEA, Brattelid T. PREPARE: guidelines for planning animal research and testing. Lab Anim. 2018;52(2):135–41.
2. Balls M. It's Time to Reconsider The Principles of Humane Experimental Technique. ATLA Altern to Lab Anim. 2020;48(1):40–6.
3. Russell WMS, Burch RL. The Principles of Humane Experimental Technique. Methuen and Co., Ltd. 1959.
4. Dondossola D, Lonati C, Battistin M, Vivona L, Zanella A, Maggioni M, et al. Twelve-hour normothermic liver perfusion in a rat model: characterization of the changes in the ex-situ bio-molecular phenotype and metabolism. Sci Rep. 2024;14(1):1–13.
5. Muller X, Schlegel A, Kron P, Eshmuminov D, Würdinger M, Meierhofer D, et al. Novel Real-time Prediction of Liver Graft Function During Hypothermic Oxygenated Machine Perfusion Before Liver Transplantation. Ann Surg. 2019;270(5):783–90.
6. Lonati C, Schlegel A, Battistin M, Merighi R, Carbonaro M, Dongiovanni P, et al. Effluent Molecular Analysis Guides Liver Graft Allocation to Clinical Hypothermic Oxygenated Machine Perfusion. Biomedicines. 2021;9(10):1444.
7. Tiranti V, Viscomi C, Hildebrandt T, Di Meo I, Mineri R, Tiveron C. Loss of ETHE1, a mitochondrial dioxygenase, causes fatal sulfide toxicity in ethylmalonic encephalopathy. Nat Med. 2009;15(2):200–5.
8. Protti A, Fortunato F, Monti M, Vecchio S, Gatti S, Comi GP, et al. Metformin overdose, but not lactic acidosis per se, inhibits oxygen consumption in pigs. Crit Care. 2012;16(3):1–8.
9. Monzio Compagnoni G, Kleiner G, Bordoni A, Fortunato F, Ronchi D, Salani S, et al. Mitochondrial dysfunction in fibroblasts of Multiple System Atrophy. Biochim Biophys Acta - Mol Basis Dis. 2018;1864(12):3588–97.
10. Bresolin N, Bet L, Moggio M, Meola G, Fortunato F, Comi G, et al. Muscle glucose-6-phosphate dehydrogenase deficiency. J Neurol. 1989;236(4):193–8.
11. Meszaros AT, Hofmann J, Buch ML, Cardini B, Dunzendorfer-Matt T, Nardin F, et al. Mitochondrial respiration during normothermic liver machine perfusion predicts clinical outcome. EBioMedicine. 2022 Nov;85:104311.
